# Conserved Senescence Associated Genes and Pathways in Primary Human Fibroblasts Detected by RNA-Seq

**DOI:** 10.1371/journal.pone.0154531

**Published:** 2016-05-03

**Authors:** S. Marthandan, M. Baumgart, S. Priebe, M. Groth, J. Schaer, C. Kaether, R. Guthke, A. Cellerino, M. Platzer, S. Diekmann, P. Hemmerich

**Affiliations:** 1 Leibniz-Institute on Aging—Fritz Lipmann Institute e.V. (FLI), Jena, Germany; 2 Leibniz Institute for Natural Product Research and Infection Biology—Hans-Knöll-Institute e.V. (HKI), Jena, Germany; 3 Laboratory of NeuroBiology, Scuola Normale Superiore, Pisa, Italy; University of Jaén, SPAIN

## Abstract

Cellular senescence correlates with changes in the transcriptome. To obtain a complete view on senescence-associated transcription networks and pathways, we assessed by deep RNA sequencing the transcriptomes of five of the most commonly used laboratory strains of human fibroblasts during their transition into senescence. In a number of cases, we verified the RNA-seq data by real-time PCR. By determining cellular protein levels we observed that the age-related expression of most but not all genes is regulated at the transcriptional level. We found that 78% of the age-affected differentially expressed genes were commonly regulated in the same direction (either up- or down-regulated) in all five fibroblast strains, indicating a strong conservation of age-associated changes in the transcriptome. KEGG pathway analyses confirmed up-regulation of the senescence-associated secretory phenotype and down-regulation of DNA synthesis/repair and most cell cycle pathways common in all five cell strains. Newly identified senescence-induced pathways include up-regulation of endocytotic/phagocytic pathways and down-regulation of the mRNA metabolism and the mRNA splicing pathways. Our results provide an unprecedented comprehensive and deep view into the individual and common transcriptome and pathway changes during the transition into of senescence of five human fibroblast cell strains.

## Introduction

Normal human fibroblasts have a finite proliferative capacity in culture, a phenomenon termed “senescence” [1, 2]. Senescent cells remain metabolically active but exit the cell cycle and stop proliferating, resulting in a decreased incidence of cancer. *In vivo*, senescence may enable removal of damaged cells and support tissue remodeling by cell cycle arrest, induction of a secretory pathway which recruits immune cells, and by recruiting progenitor cells to re-populate the tissue [3]. Furthermore, senescent cells are involved in *in vivo* aging due to their adverse impact on function and renewal of stem cells [4]. Most importantly, senescent cells that accumulate during adulthood in mice negatively influence lifespan and promote age-dependent changes in several organs [5].

Some of the major factors responsible for cellular senescence include DNA damage [6–8], oxidative stress [9, 10] and other factors [6, 11–14], resulting in an induction of cyclin-dependent kinase inhibitors (CDKIs) [15]. In spite of the lack of a single specific marker for cellular senescence, there are several cell specific markers *in vitro* and *in vivo* [16–19]: increased cell size associated with high number of lysosomes, vacuoles and mitochondria, cytoskeletal changes [20], senescence associated increase in βgalactosidase activity (SA-β Gal) [16], telomere dysfunction-induced foci (TIF) [21, 22], up-regulation of specific cell cycle regulators [23, 24], development of senescence associated heterochromatin foci (SAHF) [17, 25], altered expression pattern of genes [26, 27], secretion of proteins associated with senescence-associated secretory phenotype (SASP) [28–31] and accumulation of Annexin V at the nuclear envelope [32]. Senescent cells, despite their viability and active metabolism, have been demonstrated to be resistant to mitogenic or apoptotic stimuli [33–35].

Several mechanisms and pathways, mainly the p53-p21 and p16-pRB axes, and telomere shortening have been well documented as cellular senescence inducers [2, 18, 21, 22, 36–46]. Human fibroblasts have been routinely used to study cellular senescence [4, 28, 47, 48]. The ability of human fibroblasts to undertake a limited number of population doublings (PDs) varying from 50 to 80, depending on fibroblast cell type, until they reach a state of permanent cell cycle arrest (called “Hayflick limit”) make them an ideal model system for investigating cellular aging [1]. Cellular senescence of some types of primary mammalian cells in culture partly mirrors the mechanisms of aging *in vivo* [4, 49]. The aim of this investigation was to identify genes and pathways associated with cellular senescence by assessing the transcriptomes of five different human fibroblast strains during *in vitro* aging. We found a strong conservation of age-associated changes in the transcriptome of these five cell strains with only a minor strain-specific contribution.

## Materials and Methods

### Cell strains

Primary human fibroblasts MRC-5 (14 weeks gestation male, fibroblasts from normal lung, normal diploid karyotype), WI-38 (3 months gestation female, fibroblasts from normal lung, normal diploid karyotype), BJ (newborn male, fibroblasts from normal foreskin, normal diploid karyotype) and IMR-90 (fibroblasts from 16 weeks female fetus, lung, normal diploid karyotype) were obtained from ATCC (LGC Standards GmbH, Wesel, Germany). HFF (primary cells, *Homo sapiens*, fibroblasts from foreskin, normal diploid karyotype) cells were kind gifts of T. Stamminger (University of Erlangen, [50]).

### Cell culture

Cells were cultured as recommended by ATCC in Dulbeccos modified Eagles low glucose medium (DMEM) with L-glutamine (PAA Laboratories, Pasching, Austria), supplemented with 10% fetal bovine serum (FBS) (PAA Laboratories). Cells were grown under normal air conditions in a 9.5% CO_2_ atmosphere at 37°C. For sub-culturing, the remaining medium was discarded and cells were washed in 1 x PBS (pH 7.4) (PAA Laboratories) and detached using trypsin/EDTA (PAA Laboratories). Primary fibroblasts were sub-cultured in a 1:4 (= 2 population doublings (PDs)) or 1:2 (= 1 PD) ratio. For stock purposes, cryo-conservation of the cell strains at various PDs were undertaken in cryo-conserving medium (DMEM + 10% FBS + 5% DMSO). Cells were immediately frozen at -80°C and stored for two to three days. Afterwards, cells were transferred to liquid nitrogen for long time storage. Re-freezing and re-thawing was not performed to avoid premature senescence [51].

**Fig 1:**
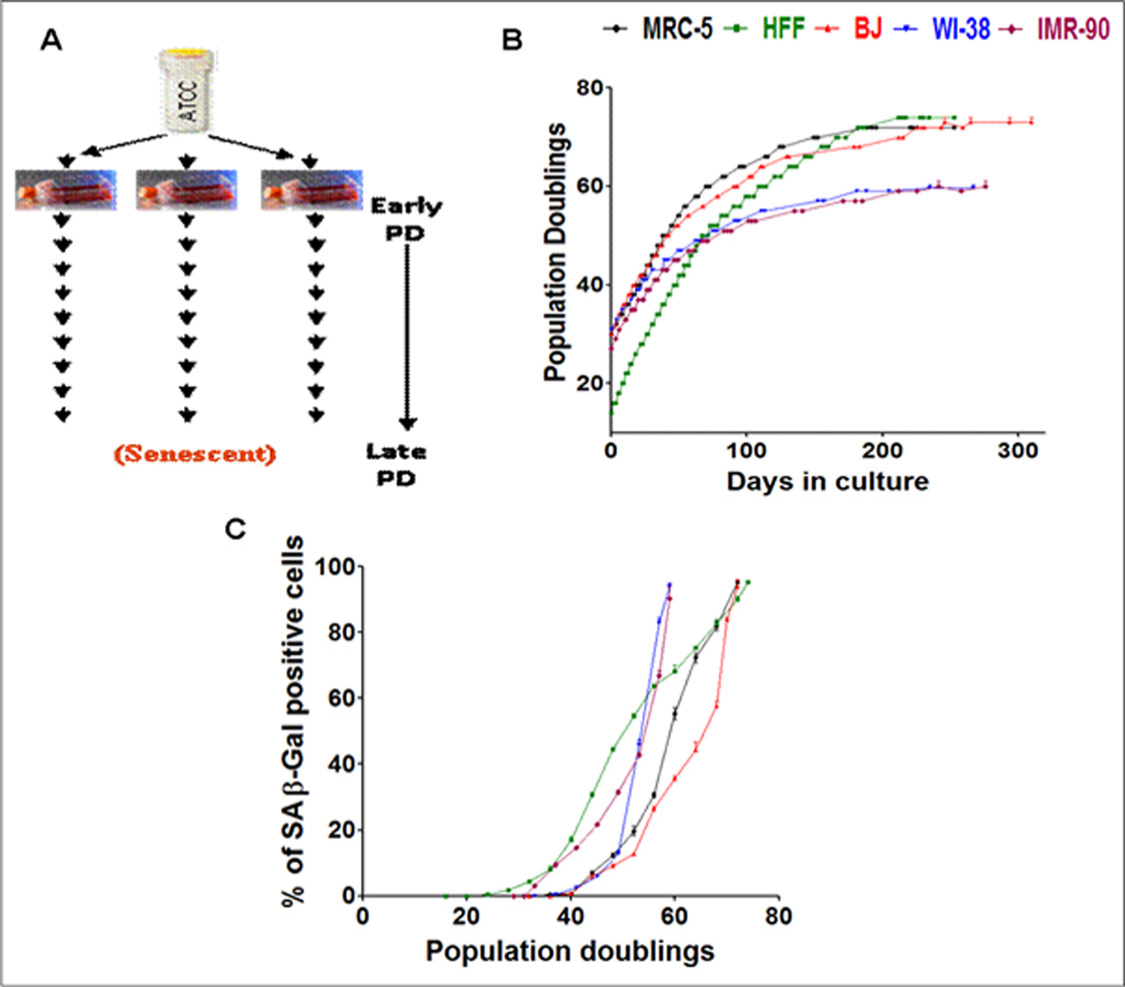
Experimental design, growth curves and transition into senescence. (A) Experimental plan of culturing fibroblast cell strains derived from a single vial and maintained in culture as triplicates from an early PD until senescence at late PDs. (B) Growth curve of five different fibroblast cell strains (MRC-5, BJ, WI-38, IMR-90, HFF) derived from a single vial and maintained in culture as triplicates from an early PD until senescence at late PDs. Data points of all measurements are displayed (not the mean). (C) Percentage of SA-β Gal positive cells at different time points of their growth in culture in the five fibroblast strains derived from a single vial. Each curve is measured in triplicate, the mean value is displayed with error bar (± S.E).

One vial of each of the five different fibroblast cell strains (MRC-5, HFF, BJ, WI-38 and IMR-90) were obtained and maintained in culture from an early PD. On obtaining enough stock on confluent growth of the fibroblasts in 75 cm^2^ flasks, cells were sub-cultured into three separate 75 cm^2^ flasks (“triplicates”) and were passaged until they were senescent in culture (Fig 1A).

### Treatment of human fibroblasts with recombinant human proteins

HFF (PD = 18), BJ (PD = 34) and MRC-5 (PD = 32) fibroblasts of early PD were maintained in conditioned DMEM medium containing different concentrations (0, 5, 10, 15, 25, 30 μg/ml) of recombinant SFRP4 (rSFRP4; 1827-SF-025; R&D SYSTEMS). SFRP4 containing medium was replenished every 2 days. A similar procedure was undertaken in HFF strains (PD = 22) with rDKK3 (1118-DK-050; R&D SYSTEMS) for a range of concentrations (0, 0.1, 1, 10, 50 μg/ml). The samples were analyzed at different time points (0, 2, 6, 10 days) for the induction of senescence using SA-β galactosidase assay (SA-β Gal) and typical senescence markers in immunoblotting.

### RNA interference and transfection method

HFF cells were transiently transfected with a range of concentrations (0–150 nM) of siRNA against SFRP4 (FlexiTube GeneSolution GS20379; Qiagen, Germany), using Lipofectamine 2000 (11668–019; Life Technologies, Invitrogen, Germany). Specific gene knock down was evaluated by immunoblotting.

### Detection of SA-β galactosidase activity

The SA-β Gal assay was performed as described by [16] in each of the five fibroblast cell strains at the frequency of every four PDs from early PDs until they were senescent (late PDs). Cells were washed in 1xPBS (pH 7.4) and fixed in 4% paraformaldehyde (pH 7.4), 10 min at room temperature (RT). After washing the cells in 1xPBS (pH 7.4), staining solution consisting of 1 mg/ml X-Gal, 8 mM citric acid /sodium phosphate pH 6,0, 5 mM K_3_Fe(CN)_6_, 5 mM K_4_Fe(CN)_6_, 150 mM NaCl, 2 mM MgCl_2_, was added. The enzymatic reaction occurred without CO_2_ for 4–16 h at 37°C. After incubation, the cells were washed in 1xPBS (pH 7.4) and, in order to visualize cell nuclei, DNA and SAHFs, mounted with 4’-6-diamidine-2-phenyl indole (DAPI) containing Prolong Gold antifade reagent (Invitrogen). Paired two-sample type 2 Student’s t-tests assuming equal variances were done to examine the values obtained from SA-β Gal assay for statistical significance.

### Immunoblotting

For immunoblotting, 10,000 cells/μl were used. Immunodetection was performed using 5%-powdered milk in PBS-T (1xPBS, pH 7.4 and 1% Tween 20) for blocking (Roth, Germany). The optimal concentration of all the primary antibodies was estimated in human fibroblasts. Primary antibodies, anti-p21 mouse antibody (OP64; Calbiochem; dilution 1:200), anti-p15 rabbit antibody (4822; Cell Signaling Technology; 1:250), anti-p16 mouse antibody (550834; BD Pharmingen; 1:200), anti-p27 rabbit antibody (sc-528; Santa Cruz; 1:200), anti-Anillin rabbit antibody (ANLN; ab99352; Abcam; 1:2000), anti-HSPC150 rabbit antibody (UBE2T; ab110459; Abcam; 1:500), anti-RNASEH2A mouse antibody (WH0010535M1; SIGMA-Aldrich; 1:500), anti-TPX2 rabbit antibody (ab71816; Abcam; 1:100), anti-KIF20A rabbit antibody (ab85644; Abcam; 1:500), anti-KIFC1 rabbit antibody (ab172620; Abcam; 1:15000), anti-CENPW rabbit antibody (PA5-34441; Thermo Scientific; 1:100), anti-RRM2 mouse antibody (ab57653; Abcam; 1:100), anti-HMGB2 rabbit antibody (ab11973; Abcam; 1:500), anti-Thymidine Kinase 1 rabbit antibody (TK1; ab76495; Abcam; 1:5000), anti-Cyclin B2 rabbit antibody (CCNB2; ab82287; rabbit; 1:100), anti-CEP55 rabbit antibody (ab84580; Abcam; 1:1000), anti-Cyclin B1 mouse antibody (CCNB1; ab72; Abcam; 1:1000), anti-Eg5 rabbit antibody (KIF11; ab61199; Abcam; 1:500), anti-Topoisomerase II alpha rabbit antibody (TOP2A; ab74715; Abcam; 1:500), anti-CDC20 rabbit antibody (ab26483; Abcam; 1:100), anti-Histone H1.2 rabbit antibody (HIST1H1C; ab17677; Abcam; 1:1000), anti-p53R2 rabbit antibody (RRM2B; ab8105; Abcam; 1:500), anti-Dkk3 goat antibody (ab2459; Abcam; 1:5000), anti-TMEM47 rabbit antibody (SAB1104840; SIGMA-Aldrich; 1:250), anti-SFRP4 rabbit antibody (ab154167; Abcam; 1:1000), anti-beta Catenin rabbit antibody (ab16051; Abcam; 1:2500) and anti-tubulin mouse antibody (T-9026; SIGMA-Aldrich; 1:5000) were diluted in 5%-powdered milk (in PBS-T) and incubated for 1 h at RT. Washing steps were performed 3×10 min in 1×PBS-T. The secondary horseradish peroxidase-labeled antibodies (Jackson Immuno Research Lab) were incubated for 1 h at RT. Detection of horseradish peroxidase was performed using ECL-detection system and radiographic film (GE Healthcare, Germany). After film development, signal intensities of immunoblot bands were quantified using Metamorph software [52]. The signal intensity values were examined for statistical significance using paired two-sample type 2 Student’s t-tests assuming equal variances.

### RNA extraction

Total RNA was isolated using Qiazol (Qiagen, Hilden, Germany) according to the manufacturer’s protocol, with modifications. In brief, the fibroblasts were pelleted in 2 ml safe-lock tubes (Eppendorf, Hamburg, Germany). 1 ml cooled Qiazol and one 5 mm stainless steel bead (Qiagen) were added. Homogenization was performed using a TissueLyzer II (Qiagen) at 20 Hz for 1 min. After incubation for 5 min at RT, 200 ml chloroform was added. The tube was shaken for 15 sec and incubated for 3 min at RT. Phase separation was achieved by centrifugation at 12,000 x g for 20 min at 4°C. The aqueous phase was transferred into a fresh cup and 10 mg of glycogen (Invitrogen, Darmstadt, Germany), 0.16 volume NaOAc (2 M, pH 4.0) and 1.1 volume isopropanol were added, mixed and incubated for 10 min at RT. The RNA was precipitated by centrifugation with 12,000 x g at 4°C for 20 min. The supernatant was removed and the pellet was washed with 80% ethanol twice and air dried for 10 min. The RNA was re-suspended in 20 μl DEPC-treated water by pipetting up and down, followed by incubation at 65°C for 5 min. The RNA was quantified with a NanoDrop 1000 (PeqLab, Erlangen, Germany) and stored at -80°C until use.

### Quantitative real-time PCR

Real-time PCR was performed by use of CFX384 thermocycler (Biorad, München, Germany) and Quantitect PCR system (Qiagen). Steps were processed as recommended by the manufacturer. We used 500 ng total RNA for cDNA synthesis in a 20 μl volume. After cDNA synthesis samples were diluted to a final volume of 200 μl with ultra-pure water. PCR reactions were performed in 10 μl volume with 2 μl diluted cDNA using the Quantitect SYBR Green PCR kit (Qiagen). A cDNA pool was serially diluted in steps of 1:1 (from 80 to 2.5 ng per reaction) and used to create standard as well as melting curves and to calculate amplification efficiencies for each primer pair prior to use for quantification. Primers are listed in S1 Table. All reactions were performed in triplicates and negative (water) as well as genomic (without reverse transcriptase) controls were always included. Fold changes describe the difference in expression level between old and young PDs normalized to three reference genes GAPDH, ACTB, and RAB10. RAB10 was selected as reference gene, since, based on our RNA sequencing (RNA-seq) results, its expression was stable through all cell strains and PDs. Statistical analysis of real-time data was performed with the relative expression software tool REST (Qiagen; [53]). This software tool uses a mathematical model which compares unknown and control samples, and calculates the significance of the differences by a pairwise fixed reallocation randomization test.

### RNA-seq

For quality check, total RNA was analyzed using Agilent Bioanalyzer 2100 (Agilent Technologies) and RNA 6000 Nano Kit (Agilent) to ensure appropriate RNA quality in terms of degradation (average RNA integrity number (RIN) of 8). Total RNA was used for Illumina library preparation and RNA-seq [54]. 2.5 μg total RNA was used for indexed library preparation using Illumina’s TruSeq RNA Sample Prep Kit v2 following the manufacturer’s instruction. Libraries were pooled and sequenced (5 samples per lane) using a HiSeq2000 (Illumina) in single read mode with 50 cycles using sequencing chemistry v3. Sequencing resulted in approximately 40 million reads with a length of 50 bp (base pairs) per sample. Reads were extracted in FastQ format using CASAVA v1.8.2 or v1.8.3 (Illumina).

### RNA-seq data analysis

Raw sequencing data were received in FASTQ format. Read mapping was performed using Tophat 2.0.6 [55] and the human genome references assembly GRCh37 (http://feb2012.archive.ensembl.org/). The resulting SAM alignment files were processed using the HTSeq Python framework and the respective GTF gene annotation, obtained from the Ensembl database [56]. Gene counts were further processed using the R programming language [57] and normalized to Reads Per Kilobase of transcript per Million mapped reads (RPKM) values. In order to examine the variance and the relationship of global gene expression across the samples, different correlation values have been computed including Spearman’s correlation of gene counts and Pearson’s correlation of log2 RPKM values. The resulting correlation values were visualized using multi-dimensional scaling plots (MDS) and heatmaps (S2 Fig).

Subsequently, the Bioconductor packages DESeq [58] and edgeR [59] were used to identify differentially expressed genes (DEG). Both packages provide statistics for determination of differential expression in digital gene expression data using a model based on the negative binomial distribution. The non-normalized gene counts have been used here, since both packages include internal normalization procedures. The resulting p-values were adjusted using the Benjamini and Hochberg’s approach for controlling the false discovery rate (FDR) [60]. Genes with an adjusted p-value < 0.05 found by both packages were assigned as differentially expressed.

### Examination of the correlation between the samples

After mapping, counting and normalization of the RNA-seq data, Spearman’s correlation was computed for each pair of samples using the expression values of all expressed genes. The resulting correlation values were visualized using Heatmap plots (S2 Fig). A similar approach had been described by [61] (S1 Fig) where the replicates exhibited high correlation since these values resulted from the re-sequencing of the same library. By this approach we determine the experimental error of our approach.

### Analysis of public data sets (previously published scientific literature on age related studies in human fibroblasts)

In order to compare our expression data of the five fibroblast cell strains with previously published gene expression data sets, we searched the Gene Expression Omnibus (GEO, http://www.ncbi.nlm.nih.gov/geo/) repository [62], the ArrayExpress Archive (http://www.ebi.ac.uk/arrayexpress; [63]) and in PubMed for studies including replicative senescence in human fibroblasts. Altogether, we found 8 studies where raw data files were available. In some cases, only a subset of all available samples was used (as indicated).

1. [64] Two-color microarray data was downloaded from GEO (GSE4352). The following samples were used: GSM85982, GSM85986, GSM85989, GSM85977, GSM85979, GSM85978, GSM85983. Early passage proliferating cells (BJ, WS1, WI-38) were compared with senescent cells.
2. [65] Two-color microarray data including two replicates was received upon request from the authors. Young and old human dermal fibroblasts (HDF) were compared.
3. [66] Two-color microarray data was downloaded from GEO (GSE687). All 16 samples from four different cell strains (human mammary stroma) were used. Proliferating cells were compared with senescent cells.
4. [67] Two-color microarray data was downloaded from GEO (GSE6762). The following samples were used: GSM155829, GSM155830, GSM155831, GSM155832. Proliferating was compared to senescent HFF strains.
5. [68] Two-color microarray data was downloaded from GEO (GSE15919). The following samples were used: GSM399555, GSM399560, GSM399561, GSM399569, GSM399571, GSM399581. Young and senescent MRC-5 fibroblasts were compared.
6. [69] Affymetrix microarray data was downloaded from GEO (GSE19018). All 12 samples were used which included young, old and senescent IMR-90 cells under either 3% or 20% oxygen (O_2_). Both O_2_ levels were analyzed independently resulting in two data sets for this study.
7. [70] Illumina Beadchip expression data was downloaded from GEO (GSE41714). This data set comprises of 12 samples from HDFs including a set of different PDs. We compared samples GSM1023041, GSM1023042, GSM1023043, GSM1023044 (early PD) with GSM1023050, GSM1023051, GSM1023052 (late PD).
8. [71] Affymetrix microarray data was downloaded from ArrayExpress (E-MTAB-2086). We compared IMR-90 samples DL10041401, DL10082501, DL10082502 (young) with DL10082507, DL10082508 (senescent).

The processing of the microarray raw data [69; 71] derived from different measurements was performed using the statistical programming language R and several R packages. The 2-color microarray [64–68] and Illumina Beadchip [70] data sets were analyzed using *GEOquery* [72], and *limma* [73]. Affymetrix microarrays [69, 71] were analyzed using *affy* [74] and custom chip-definition files downloaded from Brainarray (brainarray.mbni.med.umich.edu). Background correction, normalization, calculation of log2 fold-changes and identification of DEG was performed using *limma*. The resulting p-values were adjusted using the Benjamini and Hochberg’s approach for controlling FDR. In order to compare all data sets, gene IDs had to be converted between various formats depending on the measurement platform. We used the Ensembl database (http://www.ensembl.org/biomart/martview/) and bioDBnet (http://biodbnet.abcc.ncifcrf.gov/) as well as our own mapping pipelines.

### Retrieval of genes most significantly differentially regulated with age commonly across (and individually in) each of the five fibroblast cell strains and the public data sets

In order to retrieve the genes most significantly differentially regulated with age across all the five fibroblast cell strains and the public data sets, we applied a stringent selection criteria of p<0.05 according to both statistical packages (DESeq and edge R) and minimum RPKM of 5 in either young or old samples for the five fibroblast cell strains and p<0.05 according to *limma* for the public data sets (adjusted p-values used).

### Gene set enrichment analysis to determine the most differentially regulated pathways on aging

We used the R package *gage* [75] in order to find significantly enriched KEGG pathways. In case of our RNA-seq data the calculation was based on the gene counts and was performed as described in the methods manual. For the public microarray data sets, the calculation was based on log2 fold-changes estimated by *limma*. Estimated p-values were adjusted using the Benjamini and Hochberg’s approach for controlling false discovery rate. KEGG pathways were selected as significantly regulated if the corrected p-values were smaller than 0.05.

## Results

### Growth curve of fibroblasts and transition into senescence

We selected five different fibroblast cell strains (MRC-5, BJ, IMR-90, WI-38 and HFF) and monitored their replicative behavior during passaging into senescence. The similarities in gene expression profiles of primary human fibroblast strains derived from embryonic lung and foreskin were revealed by us previously [76]. We extended our previous study [76] with data obtained from further three fibroblast strains (BJ, IMR-90 and WI-38) in this study in order to essentially extend the statistical basis for deducing common age-driven changes in the transcriptome. In our analysis, the cell strains were derived from a single vial and were maintained in culture as triplicates from an early population doubling (PD) time point until they achieved senescence at late PDs (Fig 1A). The growth curve (Fig 1B) reveals that the starting PD of each of the fibroblasts differs according to the prehistory of the cells before arriving in our laboratory. For MRC-5 fibroblasts, the start PD was 30 for fresh vials ordered from ATCC. For BJ, IMR-90 and WI-38 the start PD was between 20 and 30. HFF cells were freshly isolated from foreskin of young boys below the age of 10 at University of Erlangen. When the HFF strain samples were received for culture, the start PD was 14. The cell strain specific transition into senescence of each of the fibroblast cell strains was detected by the induction of SA-βGal with age. The assay was performed at intervals of every four PDs (Fig 1C). The induction of senescence was earliest in HFF and IMR-90 strains (Fig 1C) during their span in culture compared to the other three fibroblast cell strains while SA-β Gal increase was late for BJ fibroblasts. Indeed, BJ fibroblasts showed the most extended replicative lifespan (Fig 1B). IMR-90 and WI-38 fibroblasts, both derived from female lung, had the least cumulative PDs approaching replicative senescence much earlier than the other fibroblast cell strains (Fig 1B and 1C). Cell strain specific differences in growth and transition into senescence were reported and discussed by us before in a quantitative study [48]. Reassuringly, the growth curves of the fibroblast strains in our study are very similar to previously undertaken studies on fibroblast strains [48, 77, 78, 79]. In particular the growth curve we obtained for HFF is almost identical to the one obtained for HDFs in a previous study of gene expression profiles of replicative senescence [70].

For each of the five different fibroblast cell strains we defined three individual time points in each growth curve: young cells (early PD) at the beginning of the linear growth curve, old cells (late PD) at the final growth stage close to senescence, and middle aged cells (mid PD) half way between the two. Since WI-38 and IMR-90 cells reached senescence at considerably lower PDs (around 60 PDs) than MRC-5, HFF and BJ cells (between 72 and 76 PDs) (Fig 1B), the PD values for young, middle and old cells differ. For the five fibroblast cell strains, the transition into senescence measured by the percentage of SA-β Gal positive cells, analyzed at young, middle and old stages of their lifespan, revealed a low standard deviation among the three parallel experiments of the triplicates (data not shown, see Materials and Methods), indicating that the observed differences in growth and transition into senescence are due to cell strain-specific properties and not due to experimental errors, consistent with our previous observations [48].

### Correlation among the mRNA expression levels of fibroblast triplicates

Pellets of young, middle aged and old cells in triplicate were collected for the five fibroblast strains and total RNA was extracted. Among the mRNA expression levels measured by RNA-seq in the primary fibroblasts, correlations were deduced among the same fibroblast cell types depending on their age (i.e. mRNA data of low PDs were compared to those of high PDs) and among the five different fibroblast cell types.

The results revealed a significant correlation among the mRNA expression levels of the triplicates in all fibroblast cell strains. Sample clustering using multidimensional scaling (MDS) plots indicated that the expression of one late PD IMR-90 fibroblast sample (one of the three vials) showed increased variance (probably due to batch effects [80]) compared to the other two vials (S3 Fig); this outlying sample was removed from further analysis [81]. Taken together, our mRNA sequencing procedure resulted in a small technical error (mean Pearson’s correlation of 0.98 within each group of replicates, one outlier removed). Clustering of the replicates in BJ and HFF (foreskin fibroblasts) was more obvious showing a larger correlation compared to IMR-90, MRC-5 and WI-38 (embryonic lung fibroblasts) (S3 Fig).

### DEG in the five fibroblasts

The mRNA expression levels at young (low PDs) and old age (high PDs) were determined for each of the five fibroblast cell strains, and the DEG were deduced, i.e. for which mRNA expression levels significantly (p<0.001) increased or decreased with age.

### Validation of RNA sequencing results with real-time PCR

In order to validate our experimental approach, mRNA expression levels of selected genes in two fibroblast cell strains MRC-5 (human embryonic lung) and HFF (human foreskin fibroblasts) were analyzed using real-time PCR. We selected a set of genes which, in these two cell strains, were most differentially expressed with age (*CTSK*, *DKK3*, *TMEM47*, *CCNB1*, *CCNA2*, *C3*, *Wnt16*, *IGFBP2* and *CCND1*) and *p21*, expected to be significantly up-regulated with age [31, 82, 83]. We found that the results from real-time PCR correlated well in terms of the direction of regulation with the sequencing-derived mRNA expression levels (S4 Fig).

### Comparison among the two hundred most differentially expressed genes in each of the five fibroblast cell strains

**Fig 2:**
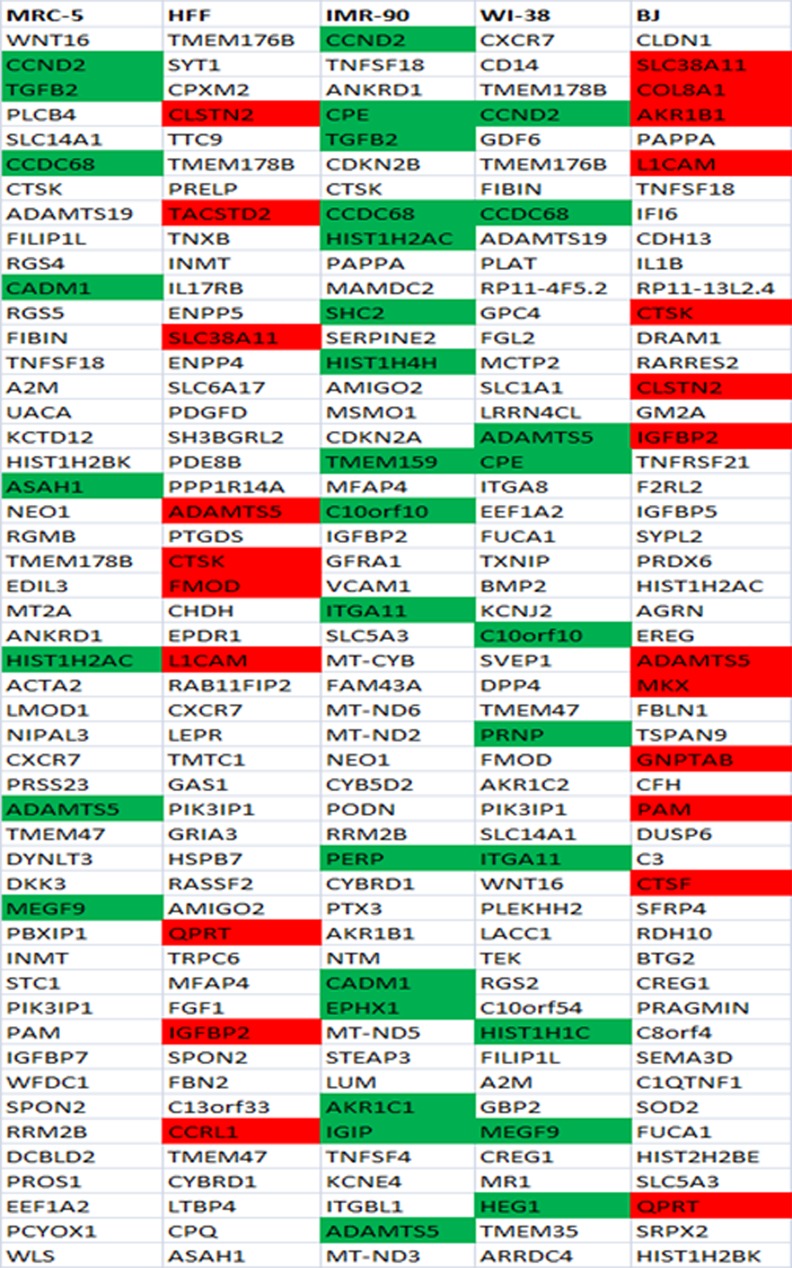
Fifty most up-regulated genes with age in each of the five fibroblast strains. The red background represents genes commonly up-regulated with age in foreskin fibroblasts (HFF, BJ) and green background reveals genes commonly up-regulated among all the three embryonic lung fibroblasts (MRC-5, IMR-90, WI-38) as well as between the fibroblasts derived from female donors (IMR-90 and WI-38) among the hundred most differentially regulated genes with age.

**Fig 3:**
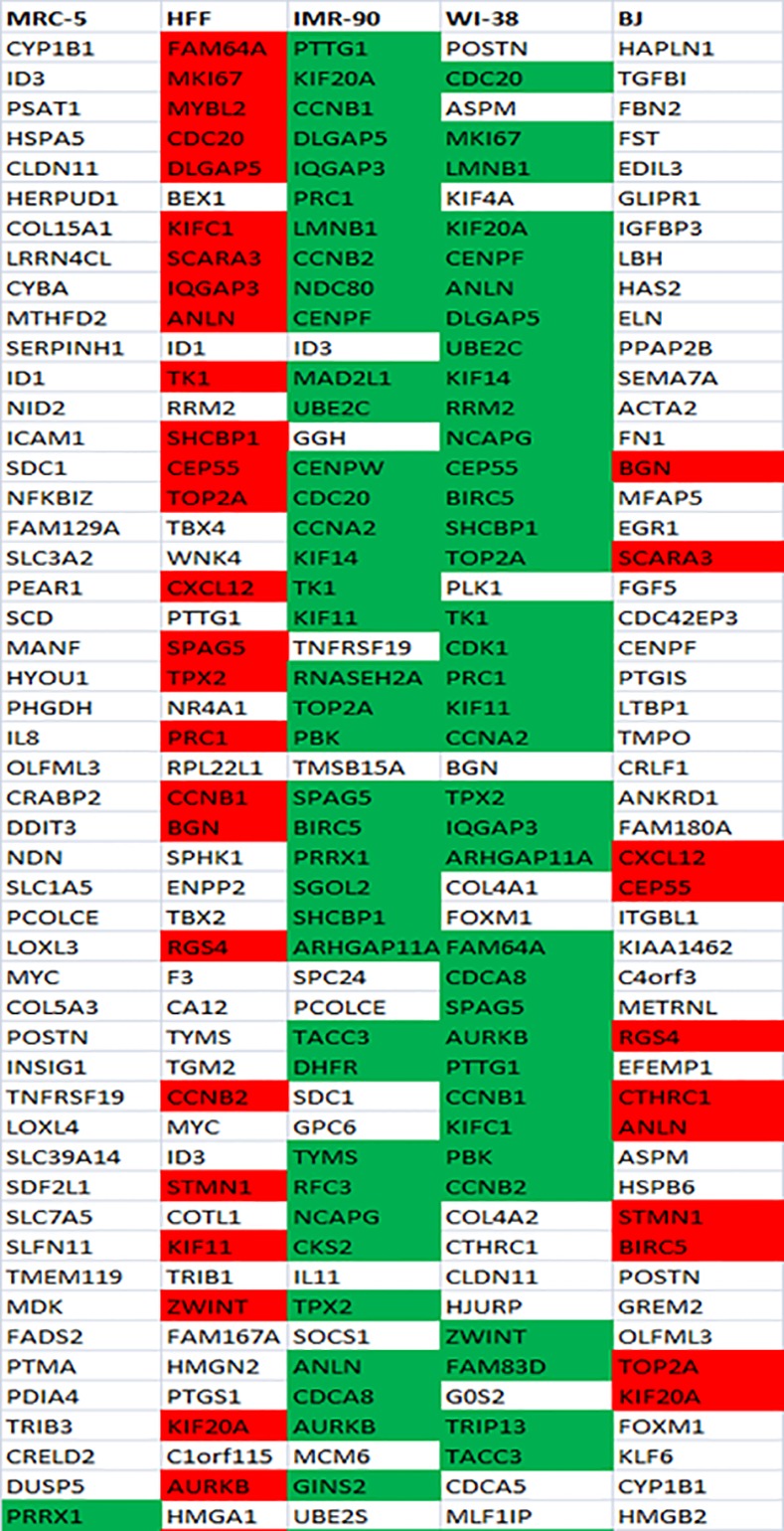
Fifty most down-regulated genes with age in each of the five fibroblast strains. The red background represents genes commonly down-regulated with age in foreskin fibroblasts (HFF, BJ) and green background reveals genes commonly down-regulated among all the three embryonic lung fibroblasts (MRC-5, IMR-90, WI-38) as well as between the fibroblasts derived from female donors (IMR-90 and WI-38) among the hundred most differentially regulated genes with age.

First, for each of the five fibroblast cell strains individually, we listed and analyzed the one-hundred most differentially up- (Fig 2, S5 Fig) and the one-hundred most differentially down-regulated (Fig 3, S6 Fig) genes with age.

When comparing these most differentially regulated genes between the two foreskin fibroblast cell strains (BJ and HFF), we identified 17 commonly up- (S2 Table) and 34 commonly down-regulated genes (S4 Table). The corresponding comparison between the three embryonic lung fibroblasts (MRC-5, IMR-90 and WI-38) revealed 10 commonly up- and 8 commonly down-regulated genes (S2 and S4 Tables). Then we compared the three lung fibroblast strains pairwise: MRC-5 and IMR-90 showed 25 commonly up- and 14 down-regulated genes, MRC-5 and WI-38 revealed 30 commonly up- and 16 down-regulated genes, while WI-38 and IMR-90, with 26 up- and 60 down-regulated genes, showed the highest number of commonly differentially regulated genes (S2 and S4 Tables). The observed differences among the three human embryonic lung fibroblasts (MRC-5, WI-38 and IMR-90) may reflect differences in the gender of the donor. MRC-5 was derived from male while IMR-90 and WI-38 were derived from female lung. Interestingly, the two female fibroblast strains IMR-90 and WI-38 both not only showed the highest number of commonly differentially regulated genes but also a considerably shorter lifespan compared to the other three fibroblast cell strains (Fig 1B and 1C).

If fibroblasts from the same tissue origin would behave similarly, we would expect a higher number of commonly differentially regulated genes amongst fibroblasts from the same tissue (foreskin or lung) than between fibroblasts from different tissues. In order to test this hypothesis, we determined the commonly regulated genes between each of the foreskin fibroblasts (HFF and BJ) on the one hand and each of the embryonic lung fibroblasts (MRC-5, IMR-90 and WI-38) on the other. HFF and MRC-5 showed 16 commonly up- and 15 commonly down-regulated genes whereas HFF and IMR-90 demonstrated 11 up- and 37 down-regulated genes while HFF and WI-38 revealed 14 up- and 41 down-regulated genes (S3 and S5 Tables). BJ and MRC-5 showed 13 up- and 11 down-regulated genes while BJ and IMR-90 revealed 18 up- and 42 down-regulated genes. WI-38 and BJ showed a different set of 18 commonly up-regulated and 52 commonly down-regulated genes (S3 and S5 Tables). These numbers do not systematically differ from the number of 17 commonly up- and 34 commonly down-regulated genes between HFF and BJ fibroblasts (see above). This indicates that the observed differences with age among the commonly differentially regulated genes across the five fibroblasts do not seem to originate from the different cell sources.

Taken together, among the five analyzed fibroblast cell strains, we found common transcriptional regulation (a mean of 19% commonly up- and 32% commonly down-regulated genes) but also considerable cell strain specific differences in the set of genes most differentially expressed after transition into senescence.

### Comparison of our study in the five fibroblast strains with previously published studies on primary human fibroblast strains

We then compared our results with similar published data [28, 64–71]. None of these published data relied on RNA-seq as in our study. The highest number of commonly regulated genes was found when we compared our mRNA expression data for each of the fibroblast cell strains individually with those retrieved from HFF strains by [67]. Several of the up-regulated genes belong to the family of insulin like growth factor binding proteins. *IGFBP3*, a marker for cellular senescence [84], is up-regulated across HFF strains in our study as well as in the study conducted by [67] and in IMR-90 fibroblasts [69]. *IGFBP5*, induced during cellular senescence [85], is up-regulated across BJ fibroblasts in our study, dermal fibroblasts [65] and in HFF strains [67]. *IGFBP2*, significantly up-regulated with senescence in retinal pigment epithelial cells [86, 87], is up-regulated in both foreskin fibroblasts (HFF and BJ) in our study and in human dermal fibroblasts [70]. *HAS2*, involved in wound healing and tissue repair [88], is down-regulated in both IMR-90 in our study and in HFF strains [67]. The most differentially regulated genes with age *KIF20A*, *KIF11*, *CCNB1*, *CCNB2*, *ANLN* and *TOP2A*, identified by [67] were also among the most commonly differentially regulated genes among the five fibroblasts in our investigation. We found a high degree of similarity between common DEG among MRC-5 fibroblasts in our study and that conducted by [68], HFF strains conducted by [67] and human dermal fibroblasts conducted by [70]. A similar high number of common DEG was retrieved on separately comparing IMR-90 and WI-38 fibroblasts in our study with the HFF data of [67] and the IMR-90 data of [71]. Thus, our RNA-seq derived transcriptome signatures of replicative senescence are fully consistent with previously published data based on gene arrays.

Next we determined the common most significantly differentially regulated genes across the five fibroblast cell strains and a sub-selection of five published data sets [64, 65, 67, 68, 70]. The stringency criteria of p<0.05 according to statistical packages (DESeq and edge R), a minimum RPKM of 5 in either young or old cells for the five fibroblast cell strains, and p<0.05 according to *limma* / adj.pvalue for the public data sets were applied. This comparison resulted in 15 up- and 7 down-regulated genes. All these genes have been annotated to specific pathways (as described in http://www.genecards.org and http://www.reactome.org). The up-regulated genes included those associated with metabolism (*RRM2B*, *HEXB*, *AKR1B1*, *SMPD1*, *PEA15*), cell cycle (*CCND1*, *DYNLT3*), apoptosis (*MOAP1*, *SERINC3*) or with membrane transport and signaling, i.e SASP (*NPC2*, *ZMAT3*, *TNFRSF10D*, *LRP10*). The down-regulated genes were associated with cell cycle (*MCM6*, *CDC6*, *SET*, *RAD21*), cellular functions (*AXL*) or mRNA splicing (*HNRNPM*, *SNRPD1*).

### Common DEG with age across the five fibroblast strains

In order to identify specific senescence-associated genes, we extracted those that were most significantly commonly differentially expressed with age across all of the five fibroblast cell strains. While the most differentially expressed genes were listed and compared for every one of the five cell strains independently (as mentioned in section entitled “Comparison among the two hundred most differentially expressed genes in each of the five fibroblast cell strains”), now those genes were deduced which were most differentially expressed commonly amongst all five cell strains together, applying the selection limit p<0.05. Of the 24,357 annotated genes, 2088 protein coding genes were affected by age among the five cell strains (8.6%). From these genes, 705 were commonly up- and 915 commonly down-regulated in all five strains while 468 genes were inconsistently regulated. Thus, 78% of the age affected DEG were commonly regulated in the same direction (either up- or down-regulated) in all five fibroblast strains. This high number indicates a strong conservation of replicative senescence associated changes in the transcriptome with only a minor strain-specific contribution, consistent with our earlier findings [76].

We then implemented more stringent selection criteria: p<0.001, log2 fold change >1, and adherence with both statistical packages (DESeq and edgeR). This very stringent selection still resulted in over 500 identified genes. When increasing the stringency of selection even further (now requiring an additional stipulation of (i) mean RPKM value >5 in each of the five fibroblast cell strains and (ii) a combined mean RPKM value >5 among the five fibroblast cell strains), 18 commonly differentially regulated genes were identified among the five different fibroblast cell strains: 2 up-regulated genes (*HIST1H1C* and *RRM2B*) and 16 down-regulated genes (*ANLN*, *UBE2T*, *TPX2*, *KIF20A*, *RNASEH2A*, *CDC20*, *TOP2A*, *KIF11*, *CCNB1*, *CEP55*, *CCNB2*, *TK1*, *HMGB2*, *RRM2*, *CENPW* and *KIFC1*). A number of these genes, including *RRM2B*, *ANLN*, *KIF20A*, *TOP2A*, *KIF11*, *CCNB1* and *CCNB2*, have already been identified by analyzing the five fibroblast cells strains independently with previously published studies (section entitled “Comparison of our study in the five fibroblast strains with previously published studies on primary human fibroblast strains”). Thus, even under strongest selection criteria, a set of genes commonly associated with the aging process in all five fibroblast strains can clearly be identified, irrespective of analyzing the cell strains independently or commonly. These genes were further investigated. For these 18 genes in each of the five fibroblasts, the protein expression levels were determined quantitatively by immunoblot band intensities. For all these 18 genes, up- or down-regulation of mRNA and protein expression values correlated well during aging (Data not shown). Thus, among the five fibroblast cell strains we could identify, even under highest selection criteria, a number of genes commonly associated with the transition into senescence. These genes of the five strain comparison overlap to some extent but not completely with those genes found by us for a two strain comparison [76]. This emphasizes the importance of increasing the number of cell strains for deducing common age-driven transcriptome changes in the cell strains. Nevertheless, the overall conclusions remain unchanged.

However, surprisingly senescence associated cell cycle inhibitors p16 (*CDKN2A*), p15 (*CDKN2B*), p21 (*CDKN1A*) and p27 (*CDKN1B*) were not among the significantly differentially regulated genes with age among the fibroblast strains. These genes are usually found to be significantly up-regulated with age in fibroblast cell strains [18]. We therefore determined, in immunoblots, the protein level of these four genes for the triplicates of the five fibroblast cell strains maintained in culture from low to high PDs. The results revealed significantly higher protein levels of p16, p15, p21 and p27 with age in all five fibroblast cell strains confirming earlier findings on the role of p16 [24, 44], p21 [21, 23, 43], p27 [18, 89–91] and p15 [18, 92] in senescence induction. We found that mRNA and protein levels correlated only in some of the fibroblast strains. This selective lack of correlation of mRNA and protein expression levels has been observed before [70, 93, 94]. However, the strong up-regulation of p16 during replicative senescence in BJ fibroblasts observed here, does not agree with previous studies [95, 96]. Thus, depending on cell strain, the age-dependent protein expression of p15, p16, p21 and p27 is regulated at the transcriptional level in some but by other down-stream mechanisms in other cases.

### Commonly regulated pathways with age

#### Commonly regulated pathways among the five fibroblast strains

**Fig 4:**
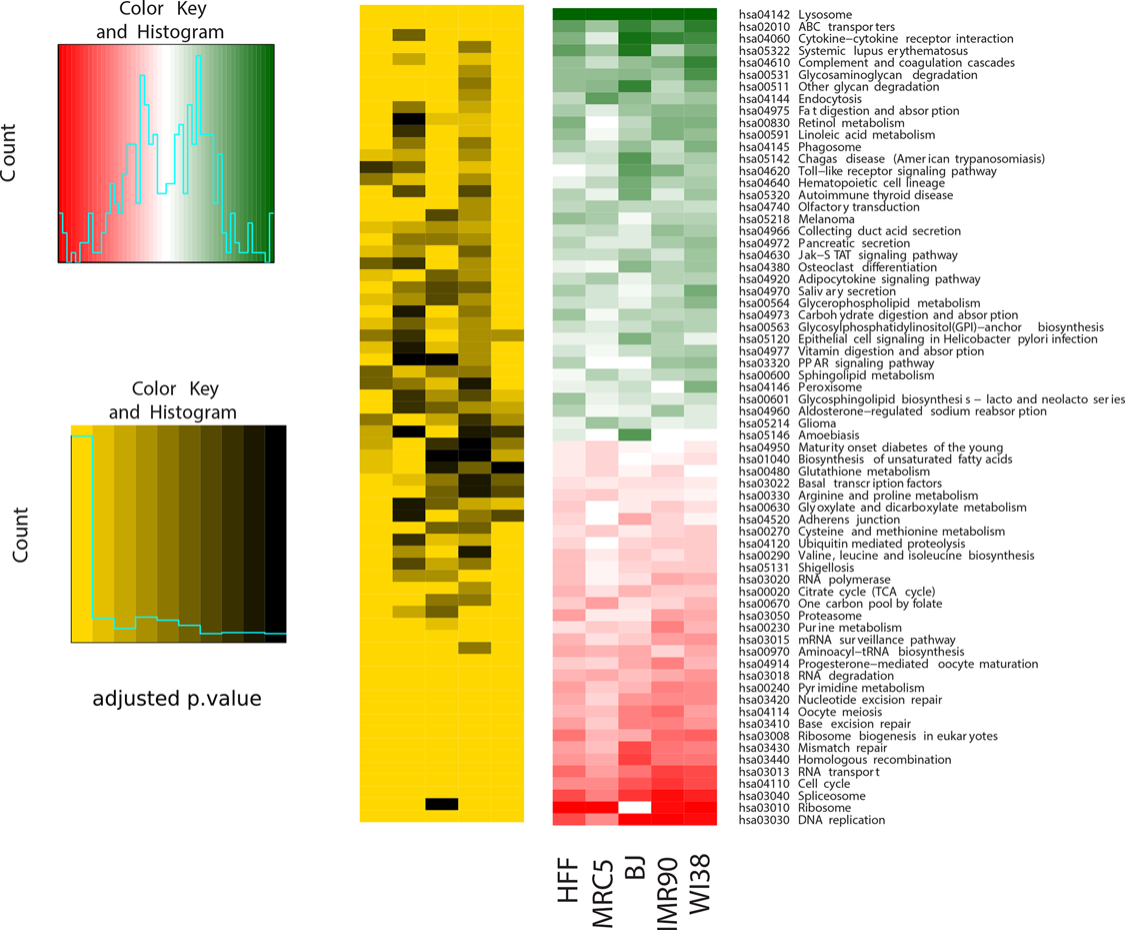
Significantly differentially regulated pathways with age across the five fibroblast strains. The most significantly up- and down-regulated pathways across the five fibroblast strains retrieved by performing gene set enrichment analysis by applying the R package *gage* (Generally Applicable Gene-set Enrichment) in combination with all annotated KEGG pathways separately for each of the five fibroblast strains.

Next, we identified the pathways most differentially up- or down-regulated with age by performing gene set enrichment analysis using the R package *GAGE* (Generally Applicable Gene-set Enrichment) in combination with all annotated KEGG pathways separately for each of the five fibroblast cell strains. The most differentially up- and down-regulated pathways in each of the five fibroblast strains are listed in S6 Table. Then, we combined all found pathways and filtered them according the same direction of regulation in all five cell strains, resulting in 36 up- and 32 down-regulated pathways (Fig 4). When applying p<0.05 as selection criteria, we found 13 pathways to be commonly down-regulated in all five cell strains, among them the well-known senescence-associated ones, such as “DNA replication (hsa03030)”, “Cell cycle (hsa04110)”, and DNA repair pathways (Fig 4), consistent with [97]. As potentially novel common down-regulated pathways we found “Spliceosome (hsa03040)”, “RNA transport (hsa03013)”, “Ribosome biogenesis (hsa03008)”, and “Pyrimidine metabolism (hsa00240)” (Fig 4). The “Spliceosome (hsa03040)” pathway is also down-regulated with age in brains of *N*. *furzeri* [98].

#### Common pathways across the five fibroblast strains and previously published datasets

Next, we compared our RNA-seq data with eight studies which, by using microarray measurements, had investigated transcriptomic changes during replicative senescence in human fibroblasts. Here, the pathways identified by us were related to pathways based on these eight studies. Since pathways group a number of genes, first we treated the transcriptome data as described below.

**Fig 5:**
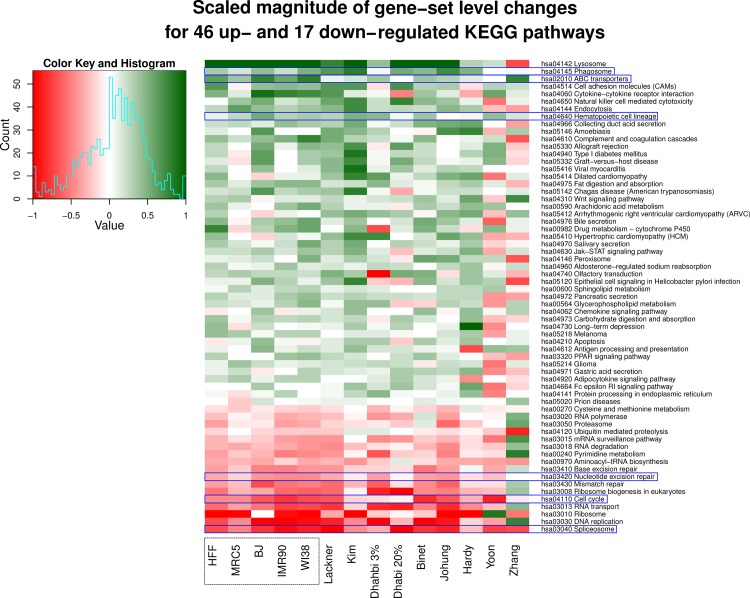
The differentially regulated pathways across the five fibroblast strains and the public data sets. The most significantly up- and down-regulated pathways across the five fibroblast strains and the nine selected public data sets retrieved by performing gene set enrichment analysis. Pathways highlighted with blue rectangles have similar regulation pattern across all the 14 datasets.

The raw data were downloaded either from GEO at NCBI or from ArrayExpress at EBI. We repeated our data analysis for the published data in order to obtain fold-changes for all measured genes available on the respective microarrays, and subsequently identified DEG using the R package *limma*. Fig 5 includes 14 different data sets: five different datasets, one for each cell strain, from this study and nine published data sets (two data sets derived from one study by [69]). Not every gene could be compared across all 14 data sets due to either missing probes on the microarray or differences within available annotation and gene ID conversion. For all comparable genes, we created pairwise scatterplots of the fold-changes, estimated their correlation and determined the number of commonly regulated genes (either up- or down-regulated with increasing PD; data not shown). For 76 out of 91 possible pairwise comparisons, more than 50% of all 1,334 comparable genes exhibited the same direction of regulation (mean = 58 ± 7%). For 79 out of the 91 comparisons, the comparable genes showed positive correlation of log2 fold-changes (mean 0.19). These results indicate both, similar regulation patterns within our data and a considerable fraction of genes with similar expression through different studies and cell strains.

We then applied gene set enrichment analysis for the nine published data sets analogous to the approach used for our five data sets. We filtered the resulting pathways for common direction of regulation for 11 out of all 14 data sets, which resulted in 46 up- and 17 down-regulated pathways (Fig 5). Notably, “Nucleotide excision repair (hsa03420)”, “Cell cycle (hsa04110)” and “Spliceosome (hsa03040)” were found again to be down-regulated in all the data sets (in Fig 5 marked with blue rectangles). As among the five fibroblast cell strains studied here, “Lysosome (hsa04142)” was one of the significantly up-regulated pathways with age among the four published data sets [67, 68, 70, 71]. From the remaining four published data sets [64–66, 68]; no significantly up-regulated pathways were obtained. The three pathways “Phagosome (hsa04145)”, “ABC transporters (hsa02010)” and “Hematopoietic cell lineage (hsa04640)” exhibit similar regulation patterns in all the data sets investigated in this study. Interestingly, the up-regulation of these three pathways with age has not been explicitly shown previously in none of the human fibroblast strains investigated in this study. However, up-regulation of expression of a number of genes associated with the regulation of the “Phagosome (hsa04145)” and “ABC transporter (hsa02010)” pathway with age was described for other cell systems [99, 100]. Furthermore, ABC transporters have been associated with providing resistance against multiple drugs in a number of cancers [101]. The up-regulation in hematopoietic cell lineage pathway with increased age was unexpected since the renewal capability of hematopoietic stem cells, originating from the hematopoietic cell lineage, decreases with age [102]. We found the same set of pathways significantly up- or down-regulated when studying two fibroblast strains (MRC-5 and HFF) [76].

Further, we undertook an in depth comparison of expression data from our five cell strains with IMR-90 microarray data from a similar study [71]. 15,278 genes were available on the microarray and included in the annotation of our RNA-seq data. IMR-90 cells from both studies behaved in a similar fashion with 74% of the genes commonly up- or down-regulated (Pearson’s correlation was 0.49 for log2 fold-changes between early and late PDs). IMR-90 cells by [71] behaved also very similar to our WI-38 cells (78% of genes with same direction of regulation; Pearson’s correlation of 0.54 for log2 fold-changes).

### Functional validation of selected genes most differentially expressed with age

Next, we investigated the expression levels of genes associated with SASP, containing proteins that were up-regulated with induction of senescence [103, 104]. As expected, a number of genes associated with SASP were significantly up-regulated with age in MRC-5 and HFF strains. The list of genes also included *IGFBP7* and *MMPs* (S4A and S4C Fig) which have been previously found to be up-regulated with senescence in other cell types [30, 104–106].

**Fig 6:**
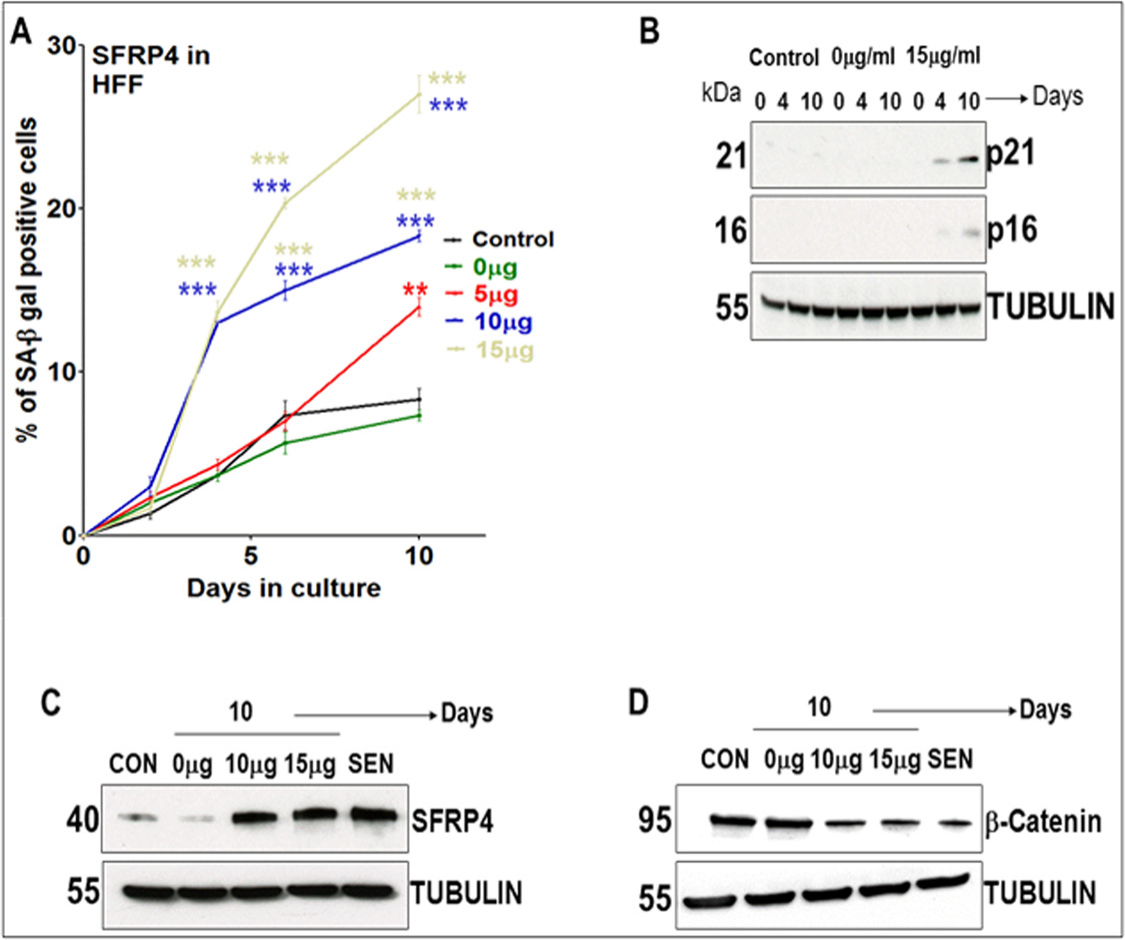
Recombinant human SFRP4 protein treatment in human foreskin fibroblasts (HFF). (A) Percentage of SA-β Gal positive cells at different time points up until 10 days in HFF strains (PD = 18) maintained in conditioned medium containing different concentrations of recombinant human SFRP4 (rSFRP4) proteins (0, 5, 10, 15 μg/ml). Samples specified control were untreated fibroblasts and those specified treated with 0 μg were treated with PBS containing 0.1% Bovine serum albumin instead of rSFRP4. The bars indicate the mean ± S.D. Values statistically different from their controls (Student’s t-test, 95% confidence level) are indicated with an asterix. ** p < 0.01, *** p <0.001- Significantly different compared to controls. n = 3 (B) Results of quantitative immunoblots showing protein expression levels of p16 and p21 in HFF strains treated with 15 μg/ml recombinant human SFRP4 proteins compared to controls and HFF strains treated with 0 μg SFRP4. n = 3 (C) Immunoblot shows protein expression levels of SFRP4 in control (CON; PD = 18), senescent (SEN; PD = 74) and young fibroblasts (PD = 18) maintained in culture supplemented with conditioned medium containing 0, 10 or 15 (μg/ml) rSFRP4 proteins for 10 days. n = 3 (D) Immunoblot shows protein expression levels of β-Catenin in control (CON; PD = 18), senescent (SEN; PD = 74) and young fibroblasts (PD = 18) maintained in culture supplemented with conditioned medium containing 0, 10 or 15 (μg/ml) rSFRP4 proteins for 10 days. n = 2.

In addition we found *SFRP4* and *DKK3*, both contributing to Wnt signaling [107, 108], significantly up-regulated with age in foreskin fibroblasts in terms of both mRNA and protein expression levels (HFF and BJ). *DKK3*, but not *SFRP4*, was also significantly up-regulated with age (both mRNA and protein levels) in embryonic lung fibroblasts (MRC-5, IMR-90, and WI-38). In order to determine whether *SFRP4* and *DKK3* have an impact on fibroblast senescence, we added recombinant SFRP4 protein to culture media of foreskin fibroblasts (HFF and BJ). We verified cellular protein up-take from the media by measuring SFRP4 protein levels in fibroblasts maintained in media containing recombinant SFRP4 proteins. Cellular protein in-take was indicated by the protein bands in immunoblots for HFF, BJ and MRC-5 cells. Treatment of HFF strains (PD = 18) with 15 μg/ml human recombinant SFRP4 protein for 10 days revealed an up-regulation of SA-β Gal in some but not all cells (26–28% of the cells, Fig 6A). p16 and p21 protein expression levels increased due to recombinant protein treatment (Fig 6B), supporting initiation of senescence induction. In order to investigate the possible antagonistic impact of SFRP4 treatment on Wnt signaling, we investigated the accumulation of β-Catenin which is functionally linked to SFRP4 [109]. β-Catenin nuclear accumulation facilitates tumor progression. β-Catenin mRNA and protein expression levels in control HFF strains decreased with age. Recombinant SFRP4 treatment down-regulated β-Catenin levels (Fig 6D), consistent with published observations [110]. With increasing amounts of recombinant human SFRP4 protein, SA-β Gal was induced (Fig 6A). Increasing SFRP4 levels in the media of young PD HFF strains further to 25 and 30 μg/ml did not induce higher SA-β Gal levels but still decreased β-Catenin levels. We obtained similar data for SFRP4 treatment in young BJ fibroblast strains (PD = 34) with 10 or 15 μg/ml. Thus, by preventing β-Catenin accumulation in HFF and BJ strains, human recombinant SFRP4 treatment (15, 25 and 30 μg/ml) may block cell proliferation. In contrast to these results obtained for HFF and BJ, adding up to 15 μg/ml recombinant SFRP4 to the culture media of embryonic lung fibroblasts (MRC-5) did neither induce SA-β Gal nor decrease β-Catenin levels. Thus, MRC-5 cells are non-responsive towards SFRP4 senescence induction signaling compared to HFF and BJ strains.

Three months after the induction of senescence (>90% SA-β Gal positive cells) in HFF and BJ fibroblast cultures, no significant changes in SFRP4 levels were observed. Since SFRP4 protein is able to initiate an induction of pre-mature senescence in young PD HFF strains (revealed by 26–28% of β Gal positive cells, Fig 6), we now asked if siRNA knock-down of SFRP4 in old (PD = 60) HFF strains (>60% SA-β Gal positive cells) can reduce the level of senescence as indicated by the number of SA-β Gal positive cells. First we verified that SFRP4 knock-down was successful. Using various concentrations ranging from 0 to 150 nM, we did not observe any effect of SFRP4 knock-down on the number of SA-β Gal positive HFF cells and on β-Catenin protein expression levels.

Then, we studied the influence of DKK3 (“dickkopf”) levels on senescence. We asked whether exogenous administration of DKK3 may initiate signaling leading to senescence induction. We thus supplemented young HFF (PD = 22) strains with recombinant DKK3 proteins (0–50 μg/ml). However, there was no effect on senescence induction. HFF strains were chosen for recombinant DKK3 protein treatment because these fibroblasts were the most sensitive to recombinant SFRP4 treatment. These experiments suggest that SFRP4 and DKK3 have no well-defined common impact on senescence induction in the fibroblast strains studied here.

## Discussion

Here we established by RNA-seq the transcriptome signature of replicative fibroblast senescence at unprecedented high resolution. Our data sets are expected to be more comprehensive as the previously published studies were based on microarrays. RNA-Seq is superior in detecting low abundance transcripts, differentiating biologically critical isoforms, a broader dynamic range, and detection of more differentially expressed genes with higher fold-change. Another benefit of RNA-seq is avoidance of technical issues inherent to microarray probe performance such as cross-hybridization, non-specific hybridization and limited detection range of individual probes [111].

By comparing the RNA-seq profiles obtained for five different fibroblast strains (MRC-5, HFF, WI-38, IMR-90 and BJ) we were able to delineate fibroblast strain-specific signatures as well as the pathways common to all of them. These data are expected to serve as an important information repository for future research on cellular aging.

### Genes most differentially regulated with age individually in each of the five fibroblast cell strains

We determined the genes most differentially regulated with age individually in each of the five fibroblast cell strains. The two female lung fibroblasts showed more similarities amongst them than compared to the other three cell strains; nevertheless, the observed differences in DEG were not significantly larger between the lung and foreskin fibroblasts than between cell strains from the same source. Thus, the observed differences were not mainly due to the different cell origin. Among the five analyzed fibroblast cell strains, we found cell strain specific differences in the senescence-associated genes, however, amongst subsets of these cell strains we identified a number of genes commonly regulated during the transition into senescence.

Most previous studies on age related changes in gene expression in human fibroblasts analyzed replicative aging only in single fibroblast cell strains [28, 65–71], only [64] studied expression values in the three fibroblasts WS1, WI-38 and BJ. Comparing data of each of our five fibroblast cell strains with these published results revealed not only a considerable similarity between our data and several published expression profiles, in particular with [67], [68], [70], [71], but also revealed several new aspects.

### Genes commonly regulated among all five fibroblast strains

Across the five fibroblast cell strains, we determined the most significantly commonly differentially expressed genes with age. From the total of 24,357 annotated genes, 78% of the age affected DEG was commonly regulated in the same direction (either up- or down-regulated). We thus found a strong conservation of age-associated changes in the transcriptome with a 22% strain-specific contribution. Interestingly, we had found the transition into quiescence [35, 112] and the hormetic response to rotenone addition [113] strongly influenced by cell strain specific effects.

By applying very stringent statistical selection criteria, we revealed those genes which were most differentially regulated commonly with age among all of the five fibroblast cell strains. A number of these genes were also identified by analyzing the five fibroblast cell strains independently. The protein expression levels of these 18 genes correlated well with the mRNA expression levels of each of these genes, indicating that the expression of these proteins is regulated at the transcriptional level. However, in some cell strains, the protein expression of some CDK inhibitors was found not to be regulated solely at the transcriptional level (see section “Common DEG with age across the five fibroblast strains”). The functional annotation of these genes with software DAVID [114] revealed that most of the 18 commonly differentially regulated genes with age were cell cycle regulatory genes. Previous studies in various cell types demonstrated an association of almost all of the above genes with proliferation, cell cycle arrest or senescence [28, 115–135].

Together with the above genes, it was also interesting to note a significant down-regulation of FOXM1 mRNA expression levels across all the five fibroblast cell strains. FOXM1 is a major transcription factor having a role in cell cycle progression, mitotic division, genomic stability as well as expression of a number of G2/M phase specific genes including CENPF and CCNB2 [136, 137]. Also the mRNA expression of the transcription factor E2F1 is significantly down-regulated with age across all five fibroblast cell strains, but to a lower extent when compared to the down-regulation of FOXM1. E2F1 induces a senescent phenotype when over-expressed in normal human fibroblasts [138]. Furthermore, extracellular matrix-remodeling genes (MMP-1, Stromelysin-1^MMP-3^, and PAI-1^SERPINE1^) are also up-regulated in senescent WI-38 fibroblasts [138]. MMP-1 was significantly up-regulated with age only in HFF and WI-38 fibroblasts whereas Stromelysin-1^MMP-3^ was significantly up-regulated with age in HFF and PAI-1^SERPINE1^ in MRC-5 fibroblasts, indicating cell strain specific differences. Though we found other transcription factors including ATF1 [139, 140], HSF1 [141, 142], CREB1 [143] and NFκB1 [144] down-regulated with age among at least three of the five fibroblast cell strains, they were not down-regulated to a significant extent. Additional experiments are required in order to understand the mechanism(s) responsible for their down-regulation.

### Pathways commonly differentially regulated among the five fibroblast cell strains

We also elucidated the KEGG pathways most differentially regulated with age among the five fibroblast cell strains. Gene set enrichment analysis resulted in 36 up- and 32 significantly down-regulated pathways (Fig 4). DNA repair and cell cycle associated pathways were most prominently represented among the down-regulated pathways (Fig 4). Down-regulation of pathways associated with cell cycle progression was expected for the age-related induction of senescence. Previously we observed that the high levels of DNA damage found in old cells, were not repaired [35], explaining the oxidative DNA damage increase with age. This age-related DNA damage increase is explained properly by the down-regulation of DNA repair pathways with age [35], as found here. Among the commonly regulated pathways were some with most and some with only very few genes regulated but also mixed pathways (either with some genes up- and some down-regulated, or with some genes up-regulated in some strains but down-regulated in others). Currently we are studying in detail the genes belonging to these pathways and for future functional studies, the role of the age-dependent differentially regulated genes in pathway function will be considered. However, elucidating the mechanism responsible for the down-regulation of DNA repair and cell cycle associated pathways, and whether the down-regulation is a cause or consequence of senescence, needs further experimentation.

### Senescence associated secretory phenotype (SASP)

**Fig 7:**
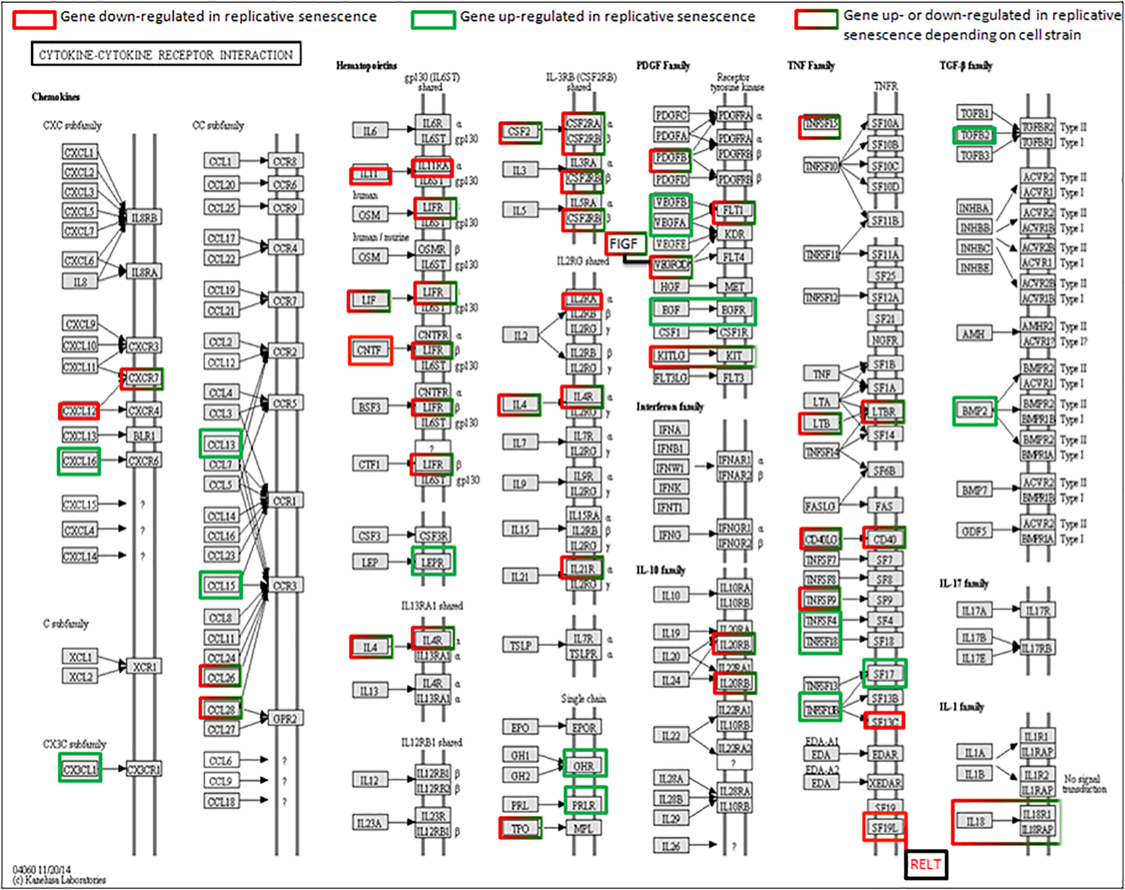
Genes belonging to the cytokine-cytokine receptor interaction pathway which are significantly up- (green) or down- (red) regulated (log 2 fold change > 1) across the five fibroblast strains during replicative senescence. Genes highlighted with boxes colored red+green are up-regulated in certain and down-regulated in the other fibroblast strains.

As expected, several genes associated with SASP were found to be most differentially regulated with age in the five fibroblast cell strains. SASP is an important hallmark and functional mediator of senescent cells [31]. In order to assess SASP expression at the transcriptional level, we visualized within the KEGG pathway "cytokin-cytokine receptor interaction" (hsa04060) the genes differentially regulated in all five fibroblast strains (Fig 7). This pathway is significantly up-regulated across all the five fibroblast strains. While, as expected, strong up-regulation was found for many cytokines and cytokine receptors (i.e. TGFB2, BMP2, CXCL16, EGF, EGFR) in all cell strains (Fig 7, marked green), the mRNA levels of many other factors within this pathway were not found to be up-regulated, or even were down-regulated (i.e. CXCL12, IL11) (Fig 7, marked green). In addition, several of the signaling factors were up- or down-regulated cell strain specifically (IL4, IL4R) (Fig 7, marked green and red). We conclude from these observations that at the transcript level SASP regulation is very heterogenous. SASP assessment may be more reliable by directly measuring protein levels. This is consistent with a detailed SASP study showing that antibody arrays provide a much more accurate assessment of the SASP signature than mRNA profiling [83]. Nevertheless, the global up-regulation of the "cytokin-cytokine receptor interaction" pathway in all fibroblast strains (Fig 7) along with the strong up-regulation of related pathways ("Cell adhesion molecules"; ABC transporters; "Endocytosis"; "Complement and coagulation cascades") in individual strains (S6 Table)) fully confirm a strong induction of the secretory phenotype in all fibroblast strains analyzed here.

## Conclusion

We conducted a comprehensive replicative aging study in five human fibroblast strains in parallel and extensively compared ours with previously published results. The complete age-dependent gene profiles were determined by RNA-seq in triplicates. This is an unbiased approach, unlike array analyses, as transcription of all genes was determined quantitatively and redundantly. We identified a high number of commonly up- and down-regulated genes as well as pathways, irrespective of analyzing the human fibroblast strains independently or commonly. For some proteins, changes with age in protein levels were not related to the corresponding changes of mRNA levels, indicating a down-stream protein expression regulation. First functional studies, conducted by us based on our data, confirmed the involvement of the secretory pathway in aging. Currently we are investigating the regulation of genes belonging to the commonly differentially regulated pathways across the five fibroblast strains as well as the proteome profile of aging primary human fibroblasts. These studies together with the transcriptome data presented here will enable an even deeper insight into the cellular aging process.

### Data deposition

All reads have been deposited in the NCBI GEO under the accession number GSE63577 and will be made available at the time of publication.

### Ethics Statement

The human fibroblast cell strains (MRC-5, IMR-90, WI-38 and BJ) used in this investigation was ordered from ATCC. A number of senescence related studies has been undertaken using these fibroblast cell strains [145–149]. HFF strains were a kind gift of Thomas Stamminger, Erlangen, Germany. The cells were generated as described previously [150].

## Supporting Information

S1 Fig Heatmap plot for Spearman correlation of “overall” gene counts between 8 samples of mouse embryonic fibroblasts (MEF) from the study by [61]. The correlation of 4 IRS-1 replicates is larger then correlation between the 4 wildtype (wt) samples. The notation “2009” and “2010” denoted the re-sequencing of one sample (technical sequencing replicate). These samples exhibit the highest correlation values (mean = 0.997). The notation “l2/l8” and “l4/l1” denote biological replicates. These samples exhibit high correlation values too (mean = 0.990). The correlation between the two conditions (IRS-1 and wt) is lower than between all kind of replicates. This situation is confirmed by the dendrogram.
(TIF)

S2 Fig Heatmap of Spearman correlation values for 30 fibroblast strains from triplicates derived from one single vial. The colors on the left side encode the different cell lines (red: BJ; blue: HFF; green: IMR-90; violet: MRC-5; orange: WI-38). The colors on top encode the PD (gray: young stage; black: old/senescent stage). Despite the group of senescent IMR-90 samples, largest correlation can be observed between the three replicates for each PD visual by tight grouping in the dendrogram.
(TIF)

S3 Fig MDS plot revealing the correlation in the mRNA expression pattern at different PDs of MRC-5, HFF, WI-38, BJ and IMR-90 fibroblast strains. The fibroblast strains were maintained in culture as triplicates from young PD till they achieved senescence. The expression pattern reveals the clustering of early PD fibroblasts (triplicates) together and is clearly separated from the triplicate cluster of late PD fibroblasts. This pattern was observed for both HFF and MRC-5 fibroblasts. The plot also reveals a clear separation in the expression pattern between the two fibroblast cell strains.
(TIF)

S4 Fig Comparison of mean fold change of mRNA expression levels of selected genes derived from real-time PCR and RNA-seq. Mean fold change of mRNA expression levels derived from real-time PCR (A1, B1, C1, D1) and RNA-seq (A2, B2, C2, D2) of selected genes (IGFBP7, CTSK, DKK3, TMEM47, CCND1, ID3, CCNB1 [C’B1], p21, CCNA2 [C’A2], C3, Wnt16 [W’16], IGFBP2 [IGF’2]) most differentially expressed with age in MRC-5 (A1, A2, B1, B2) and HFF strains (C1, C2, D1, D2). Mean fold change values of mRNA expression levels of young PD fibroblasts were normalized to 1 and compared with expression levels of old PD fibroblasts. The bars indicate the mean ± S.D. Values statistically different from their controls (Student’s t-test, 95% confidence level) are indicated with an asterix. * p < 0.05, ** p < 0.01, *** p < 0.001—Significantly different compared to young PD fibroblasts. n = 3. Note: Fig B1, D1, decrease in mRNA expression levels of ID3 with age is significant (p < 0.01) when n = 2. Fig C1, increase in mRNA expression levels of IGFBP7 with age is significant (p < 0.001) when n = 2. Fig B2, increase in mRNA expression levels of p21 with age is significant (p < 0.05) when n = 2.
(TIF)

S5 Fig The list of significantly up-regulated genes with age in each of the five fibroblast strains (51–100). The red background represents genes commonly up-regulated with age in foreskin fibroblasts (HFF and BJ) and green background reveals genes commonly up-regulated among all the three embryonic lung fibroblasts (MRC-5, IMR-90 and WI-38) as well as between the fibroblasts derived from female donors (IMR-90 and WI-38) among the hundred most differentially regulated genes with age.
(TIF)

S6 Fig The list of significantly down-regulated genes with age in each of the five fibroblast strains (51–100). The red background represents genes commonly down-regulated with age in foreskin fibroblasts (HFF and BJ) and green background reveals genes commonly down-regulated among all the three embryonic lung fibroblasts (MRC-5, IMR-90 and WI-38) as well as between the fibroblasts derived from female donors (IMR-90 and WI-38) among the hundred most differentially regulated genes with age.
(TIF)

S1 Table Forward and reverse sequence of the primers. The forward and reverse sequence of the primers designed for selected genes investigated for their mRNA expression levels using real-time PCR.
(TIF)

S2 Table Common significantly up-regulated genes classified based on origin of the fibroblast strains. List of commonly up-regulated genes with age between foreskin fibroblasts (HFF and BJ), among the embryonic lung fibroblasts (MRC-5, IMR-90 and WI-38) and between each of the embryonic lung fibroblasts among the hundred most differentially regulated genes.
(TIF)

S3 Table Common significantly up-regulated genes on individual comparison of the foreskin fibroblast strains with embryonic lung fibroblasts. List of commonly up-regulated genes with age between foreskin and embryonic lung fibroblasts (HFF and MRC-5, HFF and IMR-90, HFF and WI-38, BJ and MRC-5, BJ and IMR-90, BJ and WI-38), among the hundred most differentially regulated genes.
(TIF)

S4 Table Common significantly down-regulated genes classified based on origin of the fibroblast strains. List of commonly down-regulated genes with age between foreskin fibroblasts (HFF and BJ), among the embryonic lung fibroblasts (MRC-5, IMR-90 and WI-38) and between each of the embryonic lung fibroblasts among the hundred most differentially regulated genes.
(TIF)

S5 Table Common significantly down-regulated genes on individual comparison of the foreskin fibroblast strains with embryonic lung fibroblasts. List of commonly down-regulated genes with age between foreskin and embryonic lung fibroblasts (HFF and MRC-5, HFF and IMR-90, HFF and WI-38, BJ and MRC-5, BJ and IMR-90, BJ and WI-38), among the hundred most differentially regulated genes.
(TIF)

S6 Table Pathways most differentially regulated with age in the five fibroblast strains. The list of significantly differentially regulated [up- and down-regulated pathways] with age in each of the five fibroblast strains (A, HFF; B, MRC-5; C, BJ; D, IMR-90; E, WI-38).
(TIF)

## Abbreviations

CDKIs: Cyclin-dependent kinase inhibitors
SA: β galactosidase–Senescence associated β- galactosidase activity
TIF: Telomere dysfunction-induced foci
SAHF: Senescence associated heterochromatin foci
SASP: Senescence-associated secretory phenotype
PD: Population doublings
pRB: Phosphorylated retinoblastoma protein
HFF: Human foreskin fibroblast
DMEM: Dulbeccos modified Eagles low glucose medium
FBS: Fetal bovine serum
PBS: Phosphate buffered saline
rSFRP4: recombinant secreted frizzled-related protein 4
DKK3: Dickkopf-related protein 3
RT: Room temperature
DAPI: 4’-6-diamidine-2-phenyl indole
GAPDH: Glyceraldehyde 3-phosphate dehydrogenase
ACTB: Beta-actin
RAB10: Ras-related protein 10
RNA-seq: High-throughput RNA sequencing
RIN: RNA integrity number
RPKM: Reads Per Kilobase of transcript per Million mapped reads
MDS plots: Multi-dimensional scaling plots
DEG: Differentially expressed genes
GEO: Gene Expression Omnibus
HDF: Human dermal fibroblasts
KEGG pathways: Kyoto Encyclopedia of Genes and Genomes
CTSK: Cathepsin K
TMEM47: Transmembrane protein 47
CCNB1: Cyclin B1
CCNA2: Cyclin A2
C3: Complement 3
Wnt-16: Protein Wnt-16
IGFBP2: Insulin-like growth factor-binding protein 2
CCND1: Cyclin D1
p21: Cyclin-dependent kinase inhibitor 1 (CDKN1A)
IGFBP3: Insulin-like growth factor-binding protein 3
IGFBP5: Insulin-like growth factor-binding protein 5
HAS2: Hyaluronan synthase 2
KIF20A: Kinesin-like protein KIF20A
KIF11: Kinesin family member 11
CCNB2: Cyclin B2
ANLN: Annilin
TOP2A: DNA topoisomerase 2-alpha
RRM2B: Ribonucleoside-diphosphate reductase subunit M2 B
HEXB: Beta-hexosaminidase subunit beta
AKR1B1: Aldo-keto reductase family 1, member B1 (aldose reductase)
SMPD1: Sphingomyelin phosphodiesterase 1
PEA15: Astrocytic phosphoprotein
DYNLT3: Dynein, light chain, Tctex-type 3
MOAP1: Modulator of apoptosis 1
SERINC3: Serine incorporator 3
NPC2: Niemann–Pick type C
ZMAT3: zinc finger matrin-type 3
TNFRSF10D: Tumor Necrosis Factor Receptor Superfamily, Member 10d
LRP10: Low Density Lipoprotein Receptor-Related Protein 10
MCM6: Minichromosome Maintenance Complex Component 6
CDC6: Cell division cycle 6
SET: SET Nuclear Proto-Oncogene
RAD21: RAD21 Homolog, Nuclear Matrix Protein 1
AXL: Receptor Tyrosine kinase
HNRPM: Heterogeneous Nuclear Ribonucleoprotein M
SNRPD1: Small Nuclear Ribonucleoprotein D1 Polypeptide
HIST1H1C: Histone Cluster 1, H1c
UBE2T: Ubiquitin-Conjugating Enzyme E2T
TPX2: Targeting protein for Xklp2
RNASEH2A: Ribonuclease H2, Subunit A
CDC20: Cell division cycle 20
TK1: Thymidine kinase 1
CEP55: Centrosomal Protein 55kDa
HMGB2: High Mobility Group Box 2
RRM2: Ribonucleotide Reductase M2
CENPW: Centromere protein W
KIFC1: Kinesin Family Member C1
GAGE: Generally Applicable Gene-set Enrichment
p16: Cyclin-Dependent Kinase Inhibitor 2A
p15: Cyclin-Dependent Kinase Inhibitor 2B
p27: Cyclin-Dependent Kinase Inhibitor 1B
MMPs: Matrix metallopeptidase
FOXM1: Forkhead Box M1
CENPF: Centromere Protein F
MEFs: Mouse embryonic fibroblasts
E2F1: E2F Transcription Factor 1
PAI-1: Plasminogen activator inhibitor
ATF1: Activating transcription factor 1
HSF1: Heat Shock Transcription Factor 1
CREB1: CAMP Responsive Element Binding Protein 1
h: hours
NFκB1: NF kappa B signaling
wt: Wild type
IRS-1: Insulin receptor substrate 1
