# Supplementary figures and images for: Conserved Senescence Associated Genes and Pathways in Primary Human Fibroblasts Detected by RNA-Seq
Source: PLoS One. 2016 May 3;11(5):e0154531. doi: 10.1371/journal.pone.0154531 (PMC4854426; doi:10.1371/journal.pone.0154531)

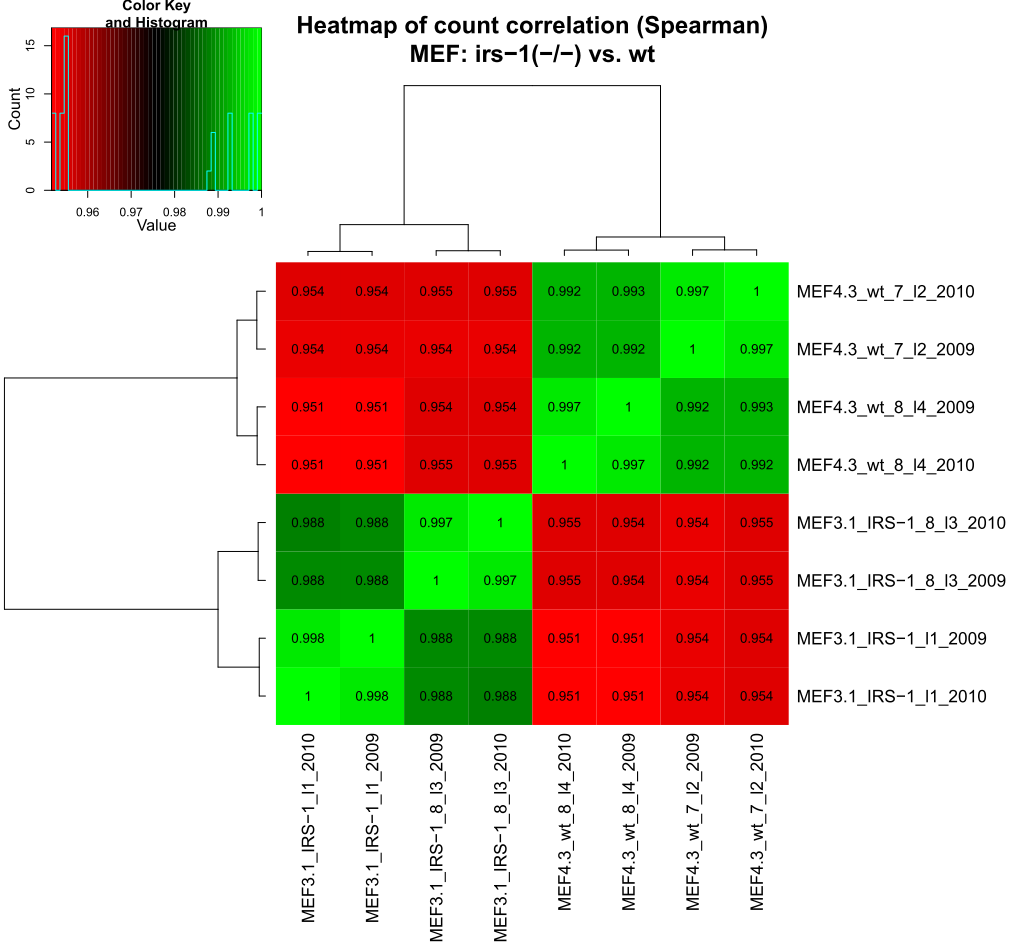

Supplement: S1 Fig — The correlation of 4 IRS-1 replicates is larger then correlation between the 4 wildtype (wt) samples. The notation “2009” and “2010” denoted the re-sequencing of one sample (technical sequencing replicate). These samples exhibit the highest correlation values (mean = 0.997). The notation “l2/l8” and “l4/l1” denote biological replicates. These samples exhibit high correlation values too (mean = 0.990). The correlation between the two conditions (IRS-1 and wt) is lower than between all kind of replicates. This situation is confirmed by the dendrogram. (TIF) [file pone.0154531.s001.tif]

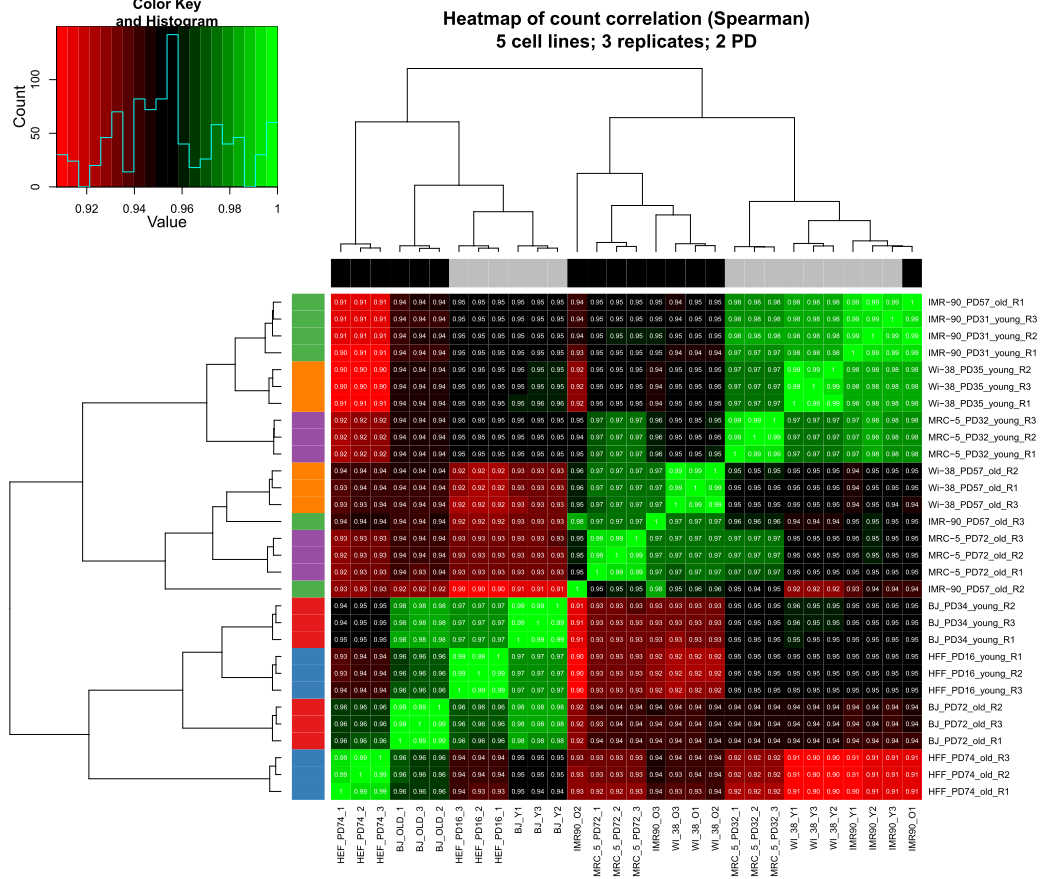

Supplement: S2 Fig — The colors on the left side encode the different cell lines (red: BJ; blue: HFF; green: IMR-90; violet: MRC-5; orange: WI-38). The colors on top encode the PD (gray: young stage; black: old/senescent stage). Despite the group of senescent IMR-90 samples, largest correlation can be observed between the three replicates for each PD visual by tight grouping in the dendrogram. (TIF) [file pone.0154531.s002.tif]

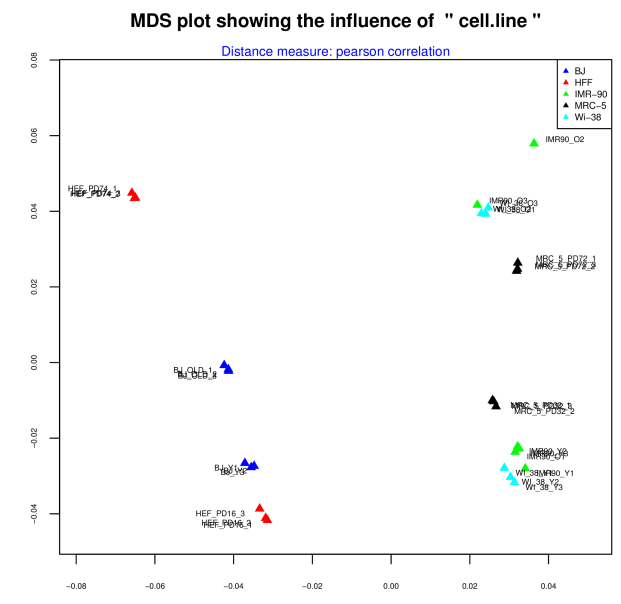

Supplement: S3 Fig — The fibroblast strains were maintained in culture as triplicates from young PD till they achieved senescence. The expression pattern reveals the clustering of early PD fibroblasts (triplicates) together and is clearly separated from the triplicate cluster of late PD fibroblasts. This pattern was observed for both HFF and MRC-5 fibroblasts. The plot also reveals a clear separation in the expression pattern between the two fibroblast cell strains. (TIF) [file pone.0154531.s003.tif]

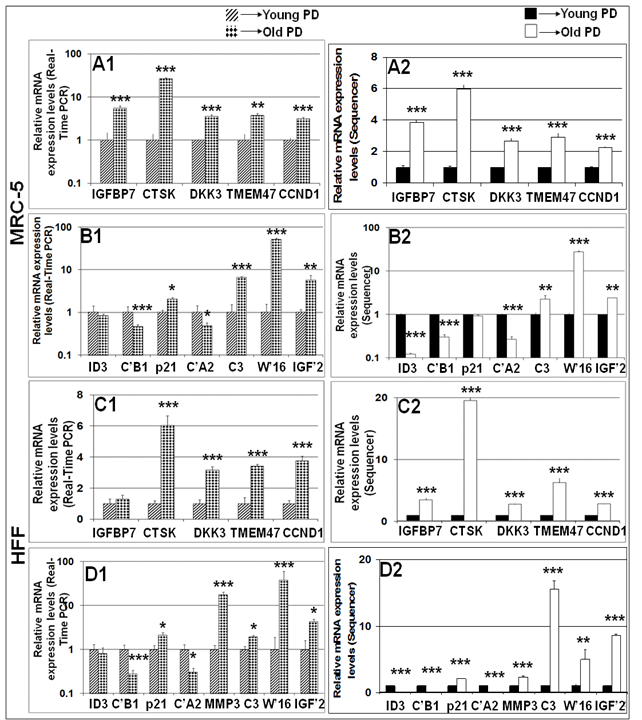

Supplement: S4 Fig — Mean fold change of mRNA expression levels derived from real-time PCR (A1, B1, C1, D1) and RNA-seq (A2, B2, C2, D2) of selected genes (IGFBP7, CTSK, DKK3, TMEM47, CCND1, ID3, CCNB1 [C’B1], p21, CCNA2 [C’A2], C3, Wnt16 [W’16], IGFBP2 [IGF’2]) most differentially expressed with age in MRC-5 (A1, A2, B1, B2) and HFF strains (C1, C2, D1, D2). Mean fold change values of mRNA expression levels of young PD fibroblasts were normalized to 1 and compared with expression levels of old PD fibroblasts. The bars indicate the mean ± S.D. Values statistically different from their controls (Student’s t-test, 95% confidence level) are indicated with an asterix. * p < 0.05, ** p < 0.01, *** p < 0.001—Significantly different compared to young PD fibroblasts. n = 3. Note: Fig B1, D1, decrease in mRNA expression levels of ID3 with age is significant (p < 0.01) when n = 2. Fig C1, increase in mRNA expression levels of IGFBP7 with age is significant (p < 0.001) when n = 2. Fig B2, increase in mRNA expression levels of p21 with age is significant (p < 0.05) when n = 2. (TIF) [file pone.0154531.s004.tif]

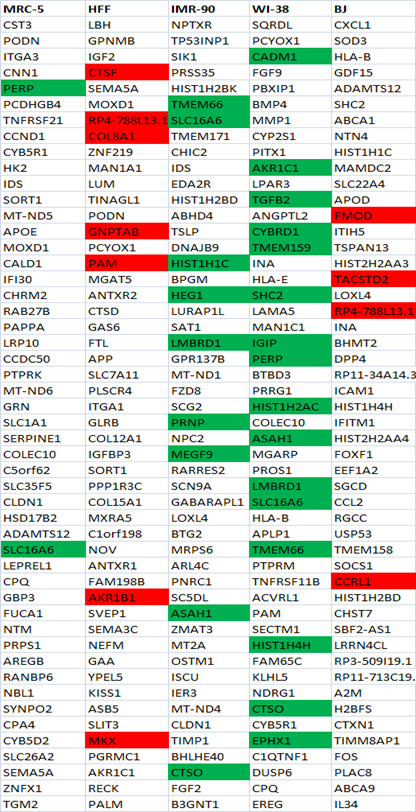

Supplement: S5 Fig — The red background represents genes commonly up-regulated with age in foreskin fibroblasts (HFF and BJ) and green background reveals genes commonly up-regulated among all the three embryonic lung fibroblasts (MRC-5, IMR-90 and WI-38) as well as between the fibroblasts derived from female donors (IMR-90 and WI-38) among the hundred most differentially regulated genes with age. (TIF) [file pone.0154531.s005.tif]

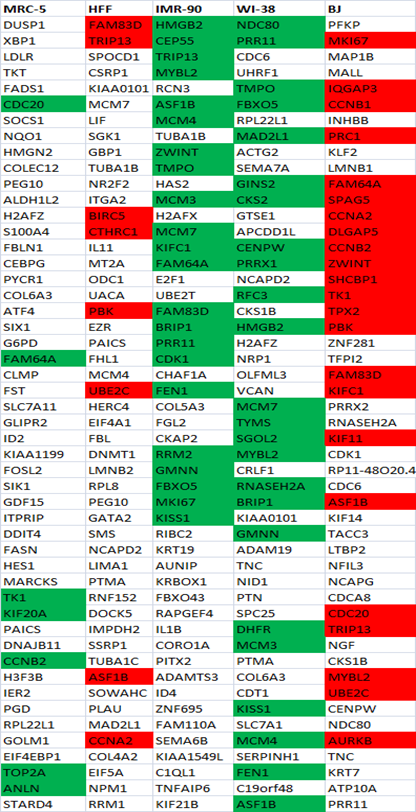

Supplement: S6 Fig — The red background represents genes commonly down-regulated with age in foreskin fibroblasts (HFF and BJ) and green background reveals genes commonly down-regulated among all the three embryonic lung fibroblasts (MRC-5, IMR-90 and WI-38) as well as between the fibroblasts derived from female donors (IMR-90 and WI-38) among the hundred most differentially regulated genes with age. (TIF) [file pone.0154531.s006.tif]

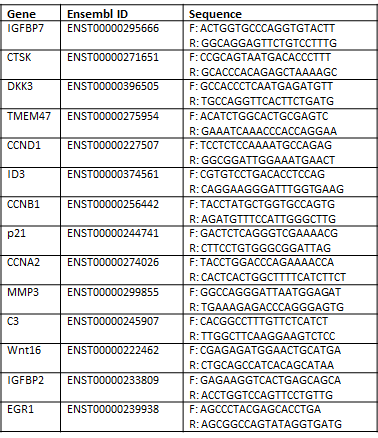

Supplement: S1 Table — The forward and reverse sequence of the primers designed for selected genes investigated for their mRNA expression levels using real-time PCR. (TIF) [file pone.0154531.s007.tif]

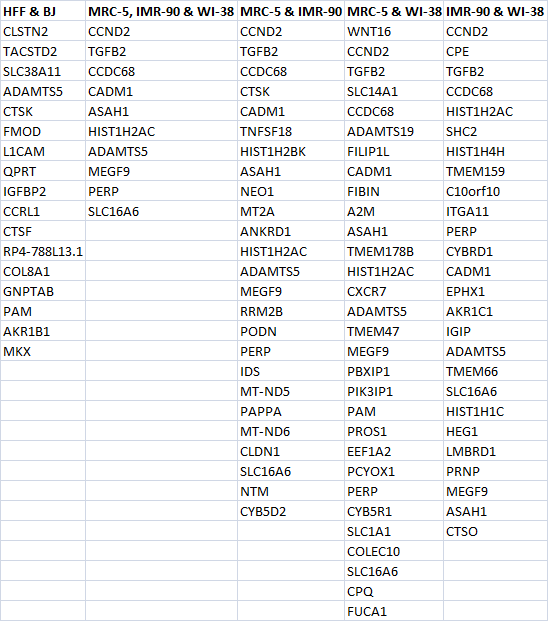

Supplement: S2 Table — List of commonly up-regulated genes with age between foreskin fibroblasts (HFF and BJ), among the embryonic lung fibroblasts (MRC-5, IMR-90 and WI-38) and between each of the embryonic lung fibroblasts among the hundred most differentially regulated genes. (TIF) [file pone.0154531.s008.tif]

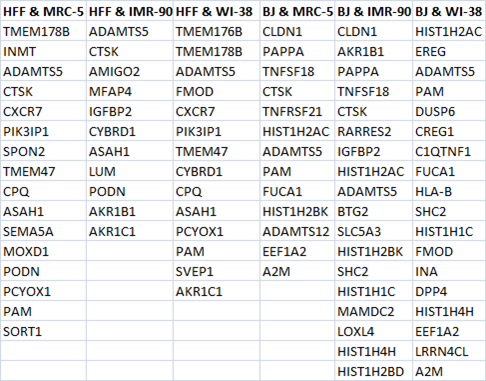

Supplement: S3 Table — List of commonly up-regulated genes with age between foreskin and embryonic lung fibroblasts (HFF and MRC-5, HFF and IMR-90, HFF and WI-38, BJ and MRC-5, BJ and IMR-90, BJ and WI-38), among the hundred most differentially regulated genes. (TIF) [file pone.0154531.s009.tif]

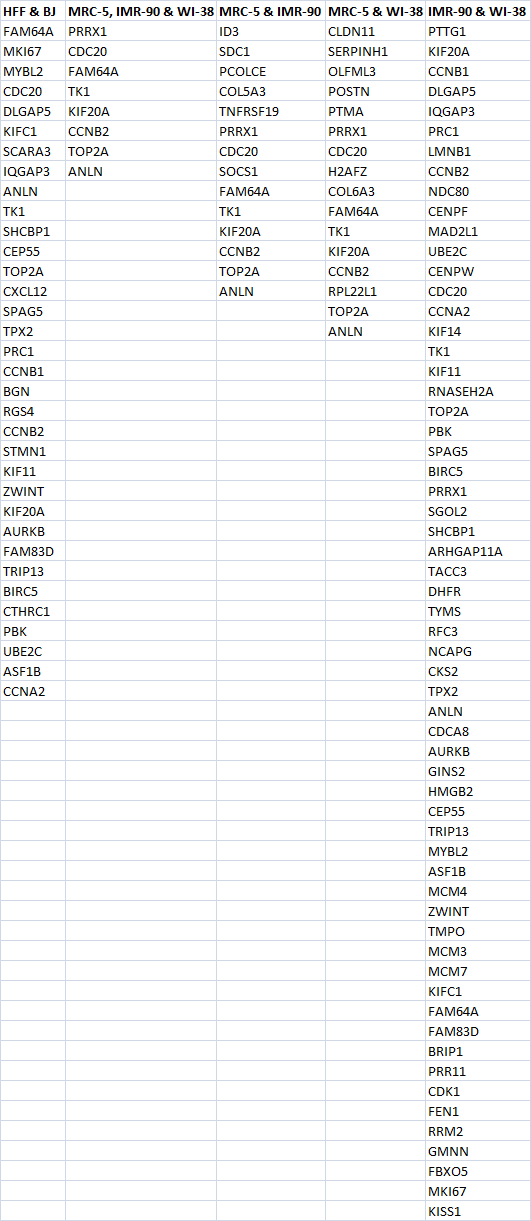

Supplement: S4 Table — List of commonly down-regulated genes with age between foreskin fibroblasts (HFF and BJ), among the embryonic lung fibroblasts (MRC-5, IMR-90 and WI-38) and between each of the embryonic lung fibroblasts among the hundred most differentially regulated genes. (TIF) [file pone.0154531.s010.tif]

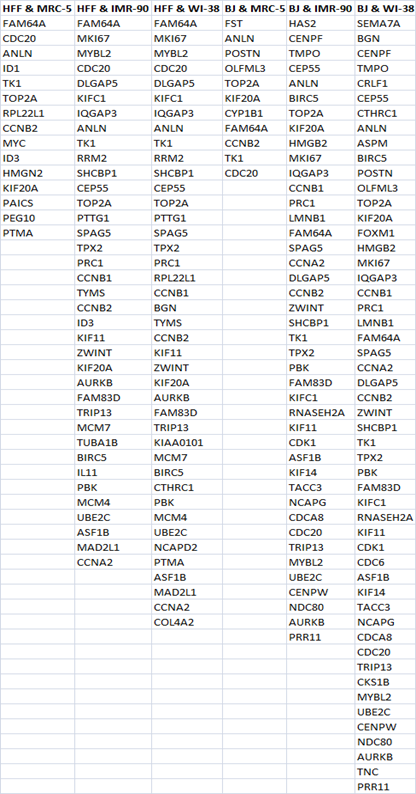

Supplement: S5 Table — List of commonly down-regulated genes with age between foreskin and embryonic lung fibroblasts (HFF and MRC-5, HFF and IMR-90, HFF and WI-38, BJ and MRC-5, BJ and IMR-90, BJ and WI-38), among the hundred most differentially regulated genes. (TIF) [file pone.0154531.s011.tif]

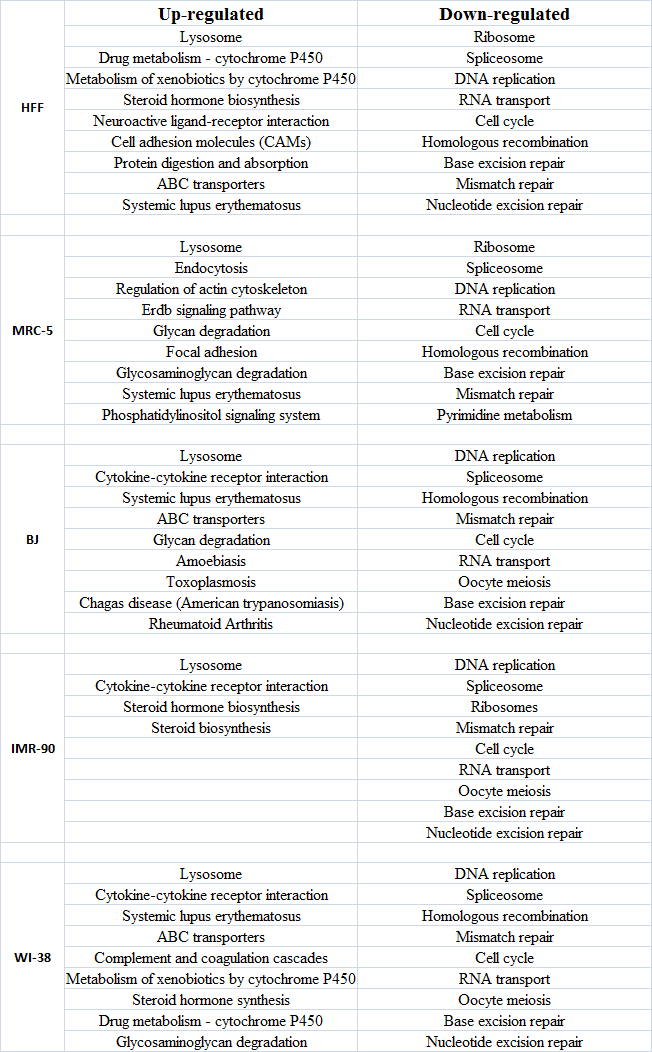

Supplement: S6 Table — The list of significantly differentially regulated [up- and down-regulated pathways] with age in each of the five fibroblast strains (A, HFF; B, MRC-5; C, BJ; D, IMR-90; E, WI-38). (TIF) [file pone.0154531.s012.tif]
